# Chemical Profiling and Photoprotective Activity of Extracts from Colombian *Passiflora* Byproducts

**DOI:** 10.3390/plants15060972

**Published:** 2026-03-21

**Authors:** María Cabeza, Cindy Lucero López, Geison Modesti Costa, Mónica Ávila-Murillo, Freddy A. Ramos, Yolima Baena, Marcela Aragón Novoa, Leonardo Castellanos

**Affiliations:** 1 Departamento de Farmacia, Facultad de Ciencias, Universidad Nacional de Colombia, Sede Bogotá, Carrera 30 # 45-03, Bogotá 11001, Colombia; mcabezap@unal.edu.co (M.C.); cillopezra@unal.edu.co (C.L.L.); ybaenaa@unal.edu.co (Y.B.); dmaragonn@unal.edu.co (M.A.N.); 2 Departamento de Química, Facultad de Ciencias, Pontificia Universidad Javeriana, Sede Bogotá, Carrera 7 # 40-62, Bogotá 110231, Colombia; 3 Departamento de Química, Facultad de Ciencias, Universidad Nacional de Colombia, Sede Bogotá, Carrera 30 # 45-03, Bogotá 11001, Colombia; mcavilam@unal.edu.co (M.Á.-M.); faramosr@unal.edu.co (F.A.R.); lcastellanosh@unal.edu.co (L.C.)

**Keywords:** *Passiflora*, byproducts, molecular networking, dereplication, GNPS, flavonoids, stilbenoids, saponins, photoprotection

## Abstract

Agro-industrial byproducts from Colombian *Passiflora* species represent an underexplored source of chemically diverse metabolites with promising cosmetic and pharmaceutical potential. This study investigated the chemical profiles and photoprotective potential of polar extracts obtained from byproducts (leaves, pericarps, and seeds) of six commercially relevant *Passiflora* species cultivated in Colombia (*P. ligularis*, *P. edulis* var. *edulis*, *P. edulis* var. *flavicarpa*, *P. maliformis*, *P. quadrangularis* and *P. tarminiana* × *P. tripartita*). Butanolic fractions from leaves and pericarps and hydroethanolic seed extracts were analyzed using ^1^H NMR, GC-FID, GC-MS and UHPLC-qTOF. NMR profiling revealed aromatic signals mainly associated with flavonoids and stilbenoids in leaves and pericarps, while seeds exhibited abundant fatty acids, particularly linoleic acid. Molecular networking enabled the visualization of chemical diversity and supported the identification of 74 metabolites, including flavonoids, saponins, and stilbenoids, using Global Natural Products Social Molecular Networking (GNPS), SIRIUS (Version 6.0.5) software, and comparison with the literature. In vitro spectrophotometric photoprotective evaluation using the Mansur equation at 200 ppm showed that leaf extracts exhibited the highest sun protection factor (SPF) values, followed by seeds and pericarps, consistent with their phenolic composition. All active extracts demonstrated broad-spectrum protection, with high UVA ratios and critical wavelength values. These findings highlight the potential of *Passiflora* byproducts as sustainable sources of natural photoprotective agents for cosmetic applications.

## 1. Introduction

The genus *Passiflora* L. is widely recognized for its remarkable chemical and biological diversity. Previous studies conducted by our research group have significantly contributed to this field by profiling flavonoids and saponins in the polar extracts of leaves from select South American *Passiflora* species, identifying key chemical markers with potential pharmaceutical applications [1,2,3]. However, given the vast diversity of *Passiflora* species in Colombia and their agro-industrial significance, further research on Colombian Passiflora species remains essential [4]. Moreover, such investigations should extend beyond leaves to include other plant parts, such as pericarps and seeds, to comprehensively assess their chemical composition and potential industrial applications [5,6].

In Colombia, several *Passiflora* species hold significant agro-industrial value, contributing to both domestic consumption and international export markets. These include yellow passion fruit or maracuyá (*Passiflora edulis* Sims var. *flavicarpa* Deg.), purple passion fruit or gulupa (*Passiflora edulis* Sims var. *edulis*), sweet granadilla or granadilla (*Passiflora ligularis* Juss), banana passion fruit or curuba (*Passiflora tarminiana* Coppens & V.E. Barney × *Passiflora tripartita* (Juss.) Poir.), sweet calabash or cholupa (*Passiflora maliformis* L.), and giant granadilla or badea (*Passiflora quadrangularis* L.) [7]. The production of these species generates considerable agro-industrial residues, primarily leaves, pericarps, and seeds, which account for approximately 60–70% of the total fruit weight [8,9]. While these byproducts are often discarded, they possess significant potential for applications in industries such as cosmetics and pharmaceuticals, where their chemical composition is essential in ensuring the safety and efficacy of final formulations [10].

Given the potential of these byproducts, it is essential to characterize their chemical composition and evaluate their possible applications. While chemical composition data are available for extracts from the leaves and seeds of certain species—particularly *P. edulis* var. *edulis* [11,12,13,14,15,16,17] and *P. edulis* var. *flavicarpa* [1,13,17,18,19,20,21,22] and, to a lesser extent, for *P. ligularis* [23,24] and *P. quadrangularis* [2,15,25]—the characterization of pericarps remains limited. This knowledge gap is especially pronounced for *P. maliformis*, for which chemical profiling studies are scarce [26]. The lack of comprehensive data on these byproducts hinders their valorization and potential industrial applications [10,27]. Therefore, systematic chemical profiling approaches are necessary to bridge this gap and unlock the full potential of these agro-industrial residues.

Chemical profiling involves the detection, identification, and/or quantification of natural compounds and includes metabolic profiling, focused on specific compound groups; and metabolic fingerprinting, an untargeted approach that detects a broader range of metabolites without necessarily achieving full identification [28,29]. These strategies commonly rely on chromatographic, spectroscopic, and mass spectrometric techniques, including hyphenated platforms such as LC-PDA, GC-FID, LC-MS, and GC-MS [30]. Within this framework, dereplication enables the recognition of previously reported metabolites based on spectroscopic and spectrometric data, allowing the analysis of complex mixtures without compound isolation [28,31]. Recent computational tools such as Global Natural Products Social Molecular Networking (GNPS) and SIRIUS have further improved dereplication workflows by facilitating the interpretation of large-scale mass spectrometric datasets and supporting molecular formula prediction, fragmentation analysis, and chemical classification [32,33,34,35,36].

In recent years, the search for natural photoprotective compounds has gained increasing attention due to the adverse effects associated with solar radiation, including sunburn, skin cancer, and photoaging [28,29,30]. Moreover, there is a growing interest in replacing conventional synthetic sunscreens with safer and more environmentally sustainable alternatives [31]. In this context, agro-industrial byproducts, such as the residues generated from the cultivation of commercial *Passiflora* species in Colombia, represent a promising source of bioactive molecules for cosmetic applications, aligned with the upcycling concept [32,33].

In this context, the present study aimed to phytochemically characterize the extracts derived from byproducts—including leaves, seeds, and pericarps—of the principal commercial *Passiflora* species in Colombia (*P. ligularis*, *P. edulis* var. *edulis*, *P. edulis* var. *flavicarpa*, *P. quadrangularis*, *P. maliformis*, and *P. tarminiana* × *P. tripartita*) using metabolomics-based approaches and to evaluate their photoprotective potential.

## 2. Results and Discussion

The extraction methods employed were selected based on previous studies, which have shown that butanolic fractions (BFs) allow the recovery of the main compounds present in *Passiflora* genus (flavonoids, saponins, phenolic compounds) from leaves and pericarps [15,34,35]. For the seeds, hydroethanolic extracts (HEs) have been reported for some *Passiflora* species, enabling the identification of various types of compounds, such as stilbenes [22]. The corresponding extraction yields (%) of BFs and HEs are presented in Table S1.

### 2.1. ^1^H NMR Preliminary Profiling

**Figure 1:**
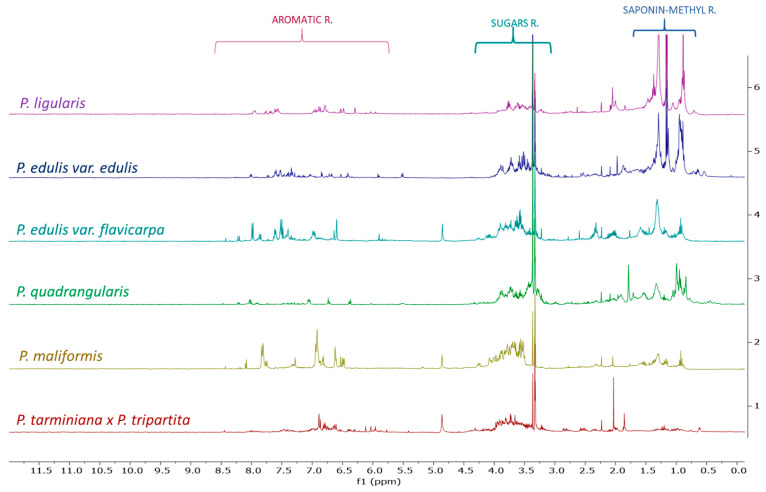
^1^H NMR spectra of the evaluated *Passiflora* BFs from leaves in MD6.

The ^1^H NMR spectra of the BFs obtained from the leaves (Figure 1) revealed three main signal regions. Characteristic signals corresponding to the aromatic protons (δ_H_ 6.0–8.0) were detected in all evaluated extracts, with higher intensity in those from *P. edulis* var. *flavicarpa*, *P. maliformis*, and *P. tarminiana* × *P. tripartita*. Additionally, signals corresponding to carbinolic protons (δ_H_ 3.5–5.5) were observed. Finally, intense signals were identified in the δ_H_ 0.75–1.25 ppm region, which were more abundant in species such as *P. ligularis*, *P. edulis* var. *edulis*, and *P. quadrangularis*.

The aromatic signals in the δ_H_ 6.0–8.0 region are characteristic of flavonoids. Previous studies have reported the presence of C-glycosylated flavonoids in *P. edulis* var. *flavicarpa* [1,19,20,36,37] and *P. tarminiana* × *P. tripartita* [38]. However, for *P. maliformis*, which also exhibited significant signal abundance in this region, no studies on leaf composition have been reported to date; only preliminary analyses of seeds and pericarps are available [39]. The signals in the δ_H_ 3.5–5.5 region are likely associated with the sugar moieties of glycosylated flavonoids, compounds characteristic of this genus [1,2,23,38]. Likewise, the intense signals observed in the δ_H_ 0.75–1.25 ppm region are characteristic of methyl groups in saponin-type compounds [40,41], aligning with previous reports on leaf extracts of *P. ligularis*, *P. edulis* var. *edulis*, and *P. quadrangularis* [15,23,42].

For the butanolic fractions (BFs) of the pericarps, the ^1^H NMR spectra predominantly exhibited signals between δ_H_ 3.0 and 4.5, while signals in the aromatic region (δ_H_ 6–8) were less prominent. The predominance of signals between δ_H_ 3.0 and 4.5 corresponds to a region typically associated with sugars and carbohydrates, which aligns with the high abundance of these compounds in this part of the fruit (Figure S1) [39,43,44]. The lower intensity of aromatic signals suggests the presence of aromatic compounds similar to those detected in the leaves, albeit at lower concentrations. Previous studies have reported the presence of flavonoids in the pericarps of several *Passiflora* species, including *P. ligularis* [45], *P. edulis* var. *edulis* [45], *P. edulis* var. *flavicarpa* [46], *P. maliformis* [39] and *P. tripartita* var. *mollisima* [45].

The ^1^H NMR spectra (Figure S2) indicate that the HEs of seeds exhibit signals at δ_H_ 2.3, 1.6, 1.29, and 1.35, as well as signals at 0.98 ppm and around δ_H_ 5.3. Additional signals were observed at δ_H_ 2.80 and 2.07. The fatty acid profile appears to be highly similar across all species. Additionally, the HEs exhibit signals in the carbohydrate region (δ_H_ 3.0–4.5) and in the aromatic region (δ_H_ 6.0–7.5), with the most distinct spectrum in the aromatic region corresponding to *P. tarminiana* × *P. tripartita*, although it still exhibits the characteristic signals observed in the other species.

The signals at δ_H_ 2.3, 1.6, 1.29, and 1.35 correspond to characteristic methylene groups of fatty acids [47]. The signal at 0.98 ppm suggests the occurrence of saturated fatty acids, while olefinic proton signals around δ_H_ 5.3 indicate the presence of double bonds, further supported by signals at δ_H_ 2.80 and 2.07 corresponding to allylic protons [42]. Given the polarity of the hydroethanolic extract, the presence of glycolipids is more likely than that of free fatty acids or monosaccharides. Moreover, the aromatic signals in the δ_H_ 6.0–7.5 region are more consistent with stilbenes than with flavonoids, as the latter typically resonate at lower field. This interpretation is consistent with previous reports of stilbenes such as piceatannol and its derivatives in *Passiflora* species, including *P. edulis* var. *flavicarpa* and *P. edulis* var. *edulis* [11,13,22,48].

### 2.2. GC-FID FAMEs Analysis

As indicated by the preliminary profiling performed via NMR and supported by previous studies, seeds from *Passiflora* species are known to contain high levels of fatty acids [49]. To further characterize these fatty acids, gas chromatography with flame ionization detection (GC-FID) and gas chromatography with mass spectrometry (GC-MS) were employed. The chromatograms of fatty acids for each species are presented in Figure S3.

The seed extracts of the evaluated *Passiflora* species exhibited low fatty acid diversity, with a largely conserved profile across species, except for *P. quadrangularis*, where two additional signals were detected, as detailed in Table 1 and Table S2. Linoleic acid was identified as the most abundant fatty acid in all species, comprising over 50% of the total fatty acids, followed by oleic and palmitic acids, while stearic acid was detected as a minor component.

This conserved fatty acid profile aligns with previously reported studies, which have extensively characterized the lipid composition of *Passiflora* seeds and confirmed the predominance of linoleic, oleic, palmitic, and stearic acids in these species [12,49,50,51,52,53,54].

In the case of *P. quadrangularis* seeds, myristic acid was uniquely identified, along with an additional signal observed between the retention times of myristic and palmitic acid. GC-MS analysis revealed a molecular ion [M^+^] with *m*/*z* 268.18 for this peak. Based on the obtained MS/MS fragmentation pattern and comparison with NIST library data, showing agreement with methyl palmitoleate, this compound was tentatively annotated as palmitoleic acid. The corresponding fragmentation spectrum is provided in Figure S4. This fatty acid has previously been reported at low concentrations in *P. edulis* seed extracts [55,56], supporting the plausibility of its occurrence in *P. quadrangularis*.

### 2.3. UHPLC-MS/MS Profiling

#### 2.3.1. Molecular Networking and Compound Classification

Molecular networks provide a graphical representation of the chemical space within analyzed extracts, enabling comparative analysis between samples and the identification of structural relationships among detected compounds [57]. Figure S5a illustrates the most representative clusters identified in negative ionization mode for the leaf BFs of the evaluated *Passiflora* species, with the ClassyFire chemical classification assigned by GNPS. This ionization mode facilitates the detection of more abundant phenolic compounds [58]. The largest clusters corresponded to flavonoids (clusters 1, 2, 5, 6, 7, and 16), followed by saponins (cluster 3) and fatty acids (clusters 4 and 27). Additionally, minor compounds were identified, including purine nucleosides (cluster 8), anthraquinones (cluster 9), growth hormones and regulators such as jasmonic acid derivatives, indole-3-acetic acid, and phenylimidazoles (clusters 12, 17, and 15), as well as alkaloid and carbazole derivatives (clusters 13 and 11).

The predominance of flavonoid clusters in negative ionization mode is consistent with the known ionization behavior of phenolic compounds and aligns with previous reports on the chemical composition of *Passiflora* leaves [15,23]. The detection of minor compound classes such as anthraquinones, alkaloids, and plant growth regulators further supports the chemical diversity previously described for this genus [59,60,61].

Among the flavonoid clusters, *P. ligularis* was predominantly enriched in flavonoid-3-*O*-glycosides (cluster 1), whereas *P. maliformis*, *P. tarminiana* × *P. tripartita*, and *P. edulis* var. *flavicarpa* showed higher abundance of flavonoid-7-*O*-glycosides (cluster 2) and flavonoid-8-*C*-glycosides (cluster 6). Regarding saponins (cluster 3), they were present in almost all species except *P. tarminiana* × *P. tripartita*, while fatty acid derivatives (cluster 4) were found across all evaluated species.

This species-dependent distribution of flavonoid subclasses is consistent with previous phytochemical studies on these taxa [2,23,24,38] and may reflect differences in glycosylation patterns within the genus. The widespread presence of fatty acid derivatives and saponins also agrees with earlier compositional analyses of *Passiflora* leaves [15,23].

The leaf BFs were further analyzed by UHPLC-MS/MS in positive ionization mode to enhance the characterization of saponins, as suggested by UHPLC-DAD-ELSD analyses. The resulting molecular network (Figure S5b) provided complementary information to the negative ionization data, facilitating the dereplication process. In this case, the most abundant clusters corresponded to steroidal glycosides (clusters 1 and 2), with chemical classification assigned by the CANOPUS tool in SIRIUS. Flavonoids (clusters 3 and 11) were also detected in this mode. Additionally, the positive ionization network revealed dipeptides (cluster 8), terpenes (clusters 6, 7, and 10), and esters (cluster 17), which were not prominently detected in negative mode.

The detection of steroidal glycosides predominantly in positive ionization mode is consistent with their enhanced ionization efficiency under these conditions. The complementary nature of both ionization modes highlights the importance of dual-mode UHPLC-MS/MS analysis for comprehensive metabolite coverage, particularly for compound classes with different ionization behaviors.

The molecular networks of the primary clusters identified in the BFs of the pericarps (Figure S6) revealed a diverse array of compounds. The most prominent clusters included *O*-glycosides, glycosylated phenolics, and flavonoid-3-*O*-glycosides (clusters 1, 2, and 3), which were widely distributed among species, particularly in *P. edulis* var. *edulis*, *P. ligularis*, *P. maliformis*, and *P. tarminiana* × *P. tripartita*. Other notable clusters comprised long-chain fatty acids (cluster 4), xanthones and purine bases (cluster 8), butenolides (cluster 5), and triterpenoids (cluster 7). Additionally, glycosylated compounds (cluster 13) were observed, though they were not exclusive to any particular species. Some clusters were species-specific, such as glycosylated iridoids and hybrid peptides, which were uniquely detected in *P. edulis* var. *edulis*.

Although a broad range of compound classes was detected, most were present in minor quantities, which is consistent with the limited chemical diversity observed during the preliminary profiling. The predominance of glycosylated phenolics and flavonoid derivatives in the major clusters agrees with previous reports describing these compounds as characteristic constituents of *Passiflora* pericarps. Furthermore, the occurrence of species-specific clusters, such as glycosylated iridoids and hybrid peptides in *P. edulis* var. *edulis*, suggests potential chemotaxonomic differences within the evaluated species.

The primary clusters identified in the seed HE networks (Figure S7) highlight stilbenoids (cluster 1) as a group of compounds present in the seeds of all evaluated species except *P. tarminiana* × *P. tripartita*. Other clusters, such as glycosylated phenolics (cluster 2), 2-arylbenzofuran flavonoids (cluster 3), and terpenes (cluster 4), were detected in most extracts. Notably, cluster 3 exhibited greater diversity in *P. quadrangularis*. Some clusters were species-specific, such as alkyl-phenyl-ketones (cluster 7) and 2′-dihydrochalcones (cluster 16), which were only found in *P. tarminiana* × *P. tripartita*.

The widespread presence of stilbenoids across species, except for *P. tarminiana* × *P. tripartita*, supports previous reports describing these compounds as characteristic constituents of *Passiflora* seeds. The higher diversity observed in cluster 3 for *P. quadrangularis* suggests a broader structural variability of 2-arylbenzofuran flavonoids in this species. Likewise, the occurrence of species-specific clusters in *P. tarminiana* × *P. tripartita* may indicate distinctive metabolic features that differentiate this taxon from the other evaluated species.

#### 2.3.2. Dereplication of Butanolic Extracts of Pericarps and Leaves of Commercial *Passiflora* Species

The UHPLC-DAD-ELSD profiles of the extracts were used to identify major compounds and establish correlations among the detected metabolites. The most abundant peaks in each evaluated extract were dereplicated using tools provided by GNPS and SIRIUS. Additionally, retention time data—specifically elution order—and UV absorption maxima (where applicable) were utilized to associate detected compounds with those previously reported in the literature or in studies from our research group. This step was essential, as some compounds have been documented in scientific reports but are not included in mass spectrometry databases. Consequently, such metabolites cannot be dereplicated solely through database searches.

For the compound dereplication process, the data were grouped by plant part (leaves, pericarps, and seeds). This decision was based on information provided by the previously mentioned tools (UHPLC-DAD-ELSD, ^1^H NMR, molecular networking), which demonstrated a greater similarity and association among compounds derived from the same plant part, regardless of species. This grouping facilitated the identification of potential common compounds across species.

When using database search engines such as GNPS and SIRIUS, it is crucial to integrate multiple tools to cross-check information and enhance reliability [59]. Additionally, the similarity scores provided by these search engines must be carefully considered. For GNPS, the cosine score is used as a mathematical measure of similarity between two fragmentation spectra (experimental and database-reported), ranging from 1 (identical fragmentation) to 0 (no similarity) [60]. For SIRIUS, the CSI:FingerID score was employed. This software predicts a molecular fingerprint for the unknown compound based on the MS/MS-derived fragmentation tree and compares it to structural fingerprints of candidate molecules retrieved from databases using a kernel-based machine learning approach. The resulting score corresponds to a log-likelihood value ranging from −∞ to 0; therefore, values closer to 0 indicate a better probabilistic agreement between the predicted and database fingerprints, rather than a conventional similarity coefficient [59,61]. For compounds identified exclusively through SIRIUS/CSI:FingerID, only candidates with a confidence score above 0.5 were considered for putative structural annotation (Schymanski Level 2) [62].

**Figure 2:**
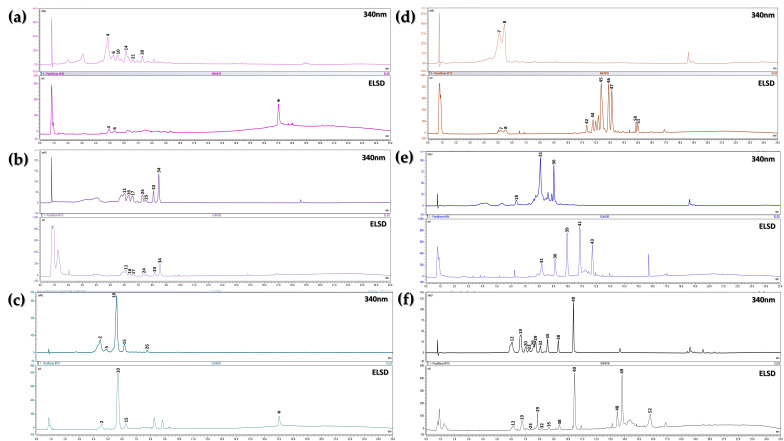
Leaf BF chromatograms, recorded at 340 nm and using an ELSD detector. Species analyzed: (**a**) *P. edulis* var. *flavicarpa*; (**b**) *P. tarminiana* × *P. tripartita*; (**c**) *P. maliformis*; (**d**) *P. quadrangularis*; (**e**) *P. edulis* var. *edulis*; (**f***) P. ligularis*. *: Signal present in the blank.

Figure 2 displays the chromatograms of the BFs from the leaves of the evaluated species, with peak numbering assigned based on retention time or elution order. The numbering for BFs from leaves and pericarps was grouped to account for compounds detected in both plant parts. Table 2 provides a comprehensive summary of the identified compounds in the BFs from leaves and pericarps, including their names, molecular ion *m*/*z* values ([M − H]^−^ or [M + H]^+^ where applicable), retention times, major fragment *m*/*z* values, UV *λmax* values (for aromatic compounds with UV absorption), the dereplication tools used (GNPS, SIRIUS, the literature, or their combination), compound type, chemical classification based on GNPS and CANOPUS (SIRIUS), mass error (ppm) relative to the exact mass reported in the literature (if identified), and similarity scores from search engines (cosine score and CSI:FingerID score).

By integrating multiple analytical approaches, we identified a total of 74 major compounds across the 12 analyzed extracts from leaves and pericarps (Table 2). The dereplication process involved assessing the confidence level of metabolite identification based on the characterization levels proposed by Schymanski and Schrimpe-Rutledge for small molecule identification using HRMS data [62,63]. Some compounds were classified as probable structure (Level 2), while others were classified at Level 3 based on chemical family, parent compound, or CANOPUS-based classification, primarily relying on fragmentation patterns. In certain cases, compounds were associated with known metabolites from the *Passiflora* genus based on shared molecular ions (Level 3). In less conclusive instances, only the molecular ion was reported (Level 4) due to the absence of comparative literature data. It is important to emphasize that dereplication was performed on complex mixtures, without isolation or structural elucidation of novel compounds. Consequently, the highest level of dereplication achieved through HRMS-based methods was Level 2.

This table highlights that flavonoids are the predominant compounds in the BFs from leaves, with glycosylated derivatives being the most abundant (31 compounds), of which 26 were identified. These are followed by saponins (13 compounds), with 10 identified, as inferred from the previously discussed molecular networks. It is important to note that while the flavonoids are present across the evaluated species and saponins in some of them, their specific chemical profiles exhibit considerable variation.

The identified flavonoids display significant structural diversity, with few occurring in only two species. For example, vitexin (10) was detected in the BFs from the leaves of *P. edulis* var. *flavicarpa*, where it had been previously reported [1], as well as in *P. maliformis*, where it was the most abundant compound despite the lack of prior documentation, likely due to the limited number of studies on this species. Conversely, the identified saponins were species-specific, with distinct profiles varying significantly among the evaluated species.

The species *P. ligularis*, *P. quadrangularis*, *and P. tarminiana* × *P. tripartita* were among those with the highest dereplication success, as they have been extensively studied [15,23,38,63]. Notably, *P. ligularis* predominantly contains *O*-glycosylated flavonoids, distinguishing it from the other species, where *C*-glycosylated flavonoids prevail. For example, *P. tarminiana* × *P. tripartita* exhibits a wide variety of *C*-glycosylated flavonoids. In contrast, *P. maliformis* is the least studied species and chemically simpler, as evidenced by fewer peaks in the DAD chromatograms. Some compounds, such as diglycosylated flavonoids (e.g., saponarin (2)) and glycosylated flavonoids (*C*-glycosylated and 7-*O*-glycosylated), were dereplicated in this species based on reports from other evaluated species, such as vitexin (10) in *P. edulis* var. *flavicarpa*.

For the BFs from the leaves of *P. edulis* var. *flavicarpa*, the extracts were rich in aromatic compounds, and some of their most abundant constituents were identified. However, lower-abundance compounds, as suggested by ELSD data, could not be identified due to insufficient MS/MS spectral quality. Additionally, the literature often lacks clarity regarding the variety of *P. edulis* evaluated, complicating the differentiation between the *flavicarpa* (yellow passion fruit) and *edulis* (purple passion fruit) varieties [36,37]. Consequently, reported compounds may not always be distinctly attributed to one variety or the other. Notably, *P. edulis* var. *flavicarpa* does not contain abundant saponins, whereas *P. edulis* var. *edulis* has.

The presence of saponin-type compounds was confirmed in *P. edulis* var. *edulis*, *P. ligularis*, and *P. quadrangularis*, with dereplication based on comparisons with previously reported data [15,23]. Interestingly, none of these compounds were dereplicated using the employed search engines, suggesting that while they are documented in the literature, they are not included in the databases integrated into these tools, thereby complicating their dereplication. Furthermore, classifications generated by GNPS and SIRIUS suggested the presence of steroidal glycosides (saponins) that have not been previously documented for the studied species. These saponins were assigned to dereplication Level 3 and represent minor components that require more comprehensive chemical investigations with larger sample quantities.

The BFs from pericarps were also analyzed using UHPLC-MS/MS. Considering the lower compound abundance compared to leaf extracts, the number of dereplicated compounds was also lower, with a total of 24 compounds identified, of which 16 were confidently assigned. It is important to note that pericarps remain one of the least studied plant parts of *Passiflora* species in terms of chemical composition. These harvest byproduct have primarily been utilized for the extraction of polymeric compounds such as pectin, a thickening agent widely used in various industries [43,44,66], and for producing flour from the pericarp of *P. edulis* var. *flavicarpa* for applications in the food industry [67]. The molecular networks of these pericarp extracts revealed the presence of glycosylated phenolics, particularly glycosylated flavonoids, which were found in relatively low abundance in these extracts. Notably, no saponins were identified in any of the studied species, in contrast to leaf extracts, where these compounds were detected and identified.

**Figure 3:**
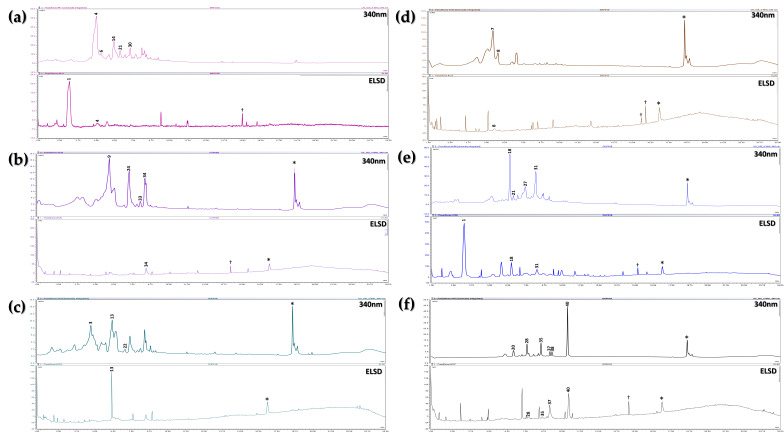
Pericarp BF chromatograms, recorded at 340 nm and using an ELSD detector. Species analyzed: (**a**) *P. edulis* var. *flavicarpa*; (**b**) *P. tarminiana* × *P. tripartita*; (**c**) *P. maliformis*; (**d**) *P. quadrangularis*; (**e**) *P. edulis* var. *edulis*; (**f***) P. ligularis*. †: Signal attributed to noise; *: Signal present in the blank.

Figure 3 presents the chromatograms of the BFs from pericarps, with identified compounds labeled and listed in Table 2, alongside the dereplicated compounds from leaf BFs. Interestingly, some dereplicated compounds from the pericarp BFs of *P. ligularis* correspond to 3-*O*-glycosylated flavonoids, a compound type that also predominates in the leaf BFs of this species. Indeed, the species with the highest compound identification rates were those with the greatest abundance and diversity of flavonoids in their leaves, such as *P. ligularis*, *P. edulis* var. *flavicarpa*, and *P. tarminiana* × *P. tripartita*. Several of these flavonoids were also detected in their pericarp BFs, consistent with previous reports [45]. Furthermore, several dereplicated compounds in these three species, such as isoorientin (4) and isovitexin (14), have already been documented in polar extracts from *P. edulis* var. *flavicarpa* pericarps [45,46,68].

In the BFs from *P. maliformis* pericarps, the identified compounds differed from those found in leaf extracts (Saponarin, Apigenin-6-*C*-arabinosyl-7-*O*-glucoside, Vitexin). These compounds have not been previously reported in the literature for the pericarps of this species. However, the detection of several flavonoids is consistent with previous preliminary phytochemical screenings [39], which reported the presence of flavonoids and other phenolic compounds. Although the dereplication rate for this species was lower compared to the other evaluated species, some flavonoids were still detected and/or tentatively identified, including schaftoside/corymboside (**3**).

#### 2.3.3. Dereplication of Major Compounds in Seeds of Commercial *Passiflora* Species

**Figure 4:**
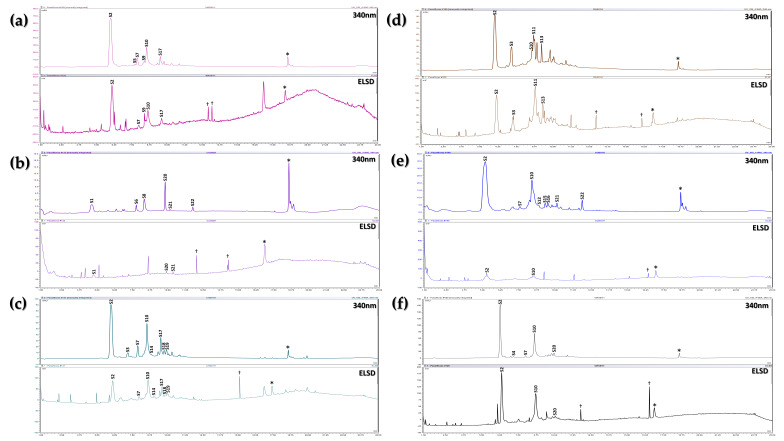
Seed HE chromatograms, recorded at 340 nm and using an ELSD detector. Species analyzed: (**a**) *P. edulis* var. *flavicarpa*; (**b**) *P. tarminiana* × *P. tripartita*; (**c**) *P. maliformis*; (**d**) *P. quadrangularis*; (**e**) *P. edulis* var. *edulis*; (**f***) P. ligularis*. †: Signal attributed to noise; *: Signal present in the blank.

The chromatograms of the HEs from the evaluated seeds are presented in Figure 4. Notably, the extracts exhibit high similarity, indicating that the detected compounds are largely shared among the species. The dereplication of abundant compounds was highly effective, resulting in the identification of 22 compounds across the six evaluated species, as detailed in Table 3.

These HEs were primarily composed of stilbenoid-type compounds, with the exception of *P. tarminiana* × *P. tripartita*, for which no stilbenoid compounds could be dereplicated. The most abundant stilbenoids were identified through GNPS as piceatannol (S1) and trans-resveratrol (S3). Additionally, minor stilbenoids were identified as dimers and trimers of these compounds, whose dereplication was performed manually by comparison with the reports of Pan et al., who previously isolated and purified these compounds from the seeds of *P. edulis* var. *flavicarpa* [48].

In addition to stilbenoids, the HEs revealed a wide diversity of flavonoids, chalcones, and other compound types. The distribution of these compounds varied among species, with P. quadrangularis seeds exhibiting the greatest diversity of minor constituents. In contrast, *P. tarminiana* × *P. tripartita* displayed a markedly different chemical profile, characterized by the absence of stilbenoids and a higher abundance of chalcones and flavonoids. Some compounds in this species were identified with a dereplication confidence level of 2, including isoliquiritigenin (S22) and catechin (S1), whereas most were assigned at levels 3 and 4 using GNPS and SIRIUS.

The high similarity among most HEs of seeds suggests that stilbenoid accumulation is largely conserved across the evaluated species. The predominance of piceatannol and trans-resveratrol, together with their oligomers, is consistent with previous reports for *P. edulis* var. *flavicarpa* seeds [48].

In contrast, the absence of stilbenoid-type compounds in *P. tarminiana* × *P. tripartita* indicates a distinct metabolic profile, which may be associated with its classification as the only representative of the Tacsonia subgenus among the studied species. Moreover, the lower dereplication confidence levels and the absence of previous reports highlight the need for more comprehensive chemical investigations to fully elucidate the metabolite profile of these seeds.

The profiling of *Passiflora* leaves, pericarps, and seeds extracts revealed significant chemical diversity. ^1^H NMR analysis identified aromatic compounds of flavonoid type, fats, and sugars in the BFs of leaves, while BFs of pericarps showed a predominance of sugar-type compounds. Seed HEs predominantly contained fats and aromatic compounds. GC-FID and GC-MS characterization of fatty acids in the seeds showed linoleic acid as the most abundant across all evaluated species, with difference in *P. quadrangularis*, which presented two additional fatty acids. Phytochemical analysis using UHPLC-MS/MS, complemented by tools such as GNPS, allowed the mapping of the chemical space of the extracts, highlighting the diversity of flavonoids and saponins in leaves and pericarps, and stilbenoids in seeds. Dereplication led to the detection of 52 compounds in the leaves and pericarps BFs, and 22 in the seed HEs, with key compounds identified as flavonoids, saponins, and stilbenoids, for the most part. These findings highlight the complexity and variability of the phytochemical profiles across the studied species and emphasize the importance of using multiple analytical tools to identify compounds in complex extracts, as some information is still not fully integrated into the search engine databases like GNPS and SIRIUS.

### 2.4. Photoprotective Activity Assesment

The initial SPF screening (Figure S8) revealed a clear influence of the plant part on photoprotective capacity. Leaves BFs showed the highest SPF values, followed by HEs of seeds and BFs of pericarps. This pattern correlates with chemical composition: leaves contained a greater diversity of phenolic compounds, mainly flavonoids, likely responsible for the activity. Seed extracts, rich in stilbenoids, may also contribute to UV absorption, whereas pericarps, being a plant part highly rich in sugars, in which the concentrations of phenolic compounds tend to be lower, exhibited a lower SPF value.

Based on the ANOVA at 100 ppm of this screening (Table S3), the six most active extracts were selected and evaluated in cell spectrophotometer: butanolic fractions (BF) from leaves of *P. edulis* var. *flavicarpa*, *P. maliformis*, and *P. tarminiana* × *P. tripartita*, and hydroethanolic extracts (HE) from seeds of *P. ligularis*, *P. edulis* var. *flavicarpa*, and *P. maliformis*. As shown in Figure S9a, SPF values determined by the Mansur method indicated that leaf BFs of *P. edulis* var. *flavicarpa* (SPF_200_ ppm = 23.27 ± 0.79), *P. maliformis* (SPF_200_ ppm = 21.89 ± 0.22), and *P. tarminiana* × *P. tripartita* (SPF_200_ ppm = 16.14 ± 0.03), together with the seed HEs of *P. ligularis* (SPF_200_ ppm = 18.96 ± 0.03), *P. maliformis* (SPF_200_ ppm = 15.51 ± 0.17), and *P. edulis* var. *edulis* (SPF_200_ ppm = 10.58 ± 0.13) showed the strongest photoprotective activity, corresponding to the high-SPF category at 200 ppm.

Leaf BFs, rich in flavonoids, exhibited the highest SPF, consistent with their phenolic diversity. This effect can be explained by the characteristic UV absorption of flavonoids, which possess conjugated aromatic systems showing two main absorption bands (Band II: 250–285 nm; Band I: 320–385 nm), directly contributing to photoprotective activity [70,71]. Reports on *Passiflora* leaf extracts photoprotective activity are limited; hydroethanolic extracts of *P. cincinnata* (Brazil) showed SPF values of 8 and 15 at 0.5% and 1% (*v*/*v*), respectively [72], while *P. foetida* (Indonesia) presented values between 11.7 and 33.9 at 2–6% [73]. Under the experimental conditions applied in the present study, comparatively high SPF values were observed at lower tested concentrations. Although methodological differences such as solvent systems and concentration ranges may influence absolute values, these results support the promising photoprotective potential of Colombian *Passiflora* species.

Seed extracts presented SPF values of 10–19, generally classified as medium protection, except for *P. ligularis* and *P. maliformis*, which reached high SPF. These findings align with their chemical profiles dominated by stilbenoids such as resveratrol and piceatannol, compounds known for their photoprotective properties [74,75,76,77]. Previous reports for *P. edulis* var. *flavicarpa* seed extracts showed SPF values of 11–18 at 1 mg/mL [78], whereas this study reached SPF = 13.8 at only 0.2 mg/mL under the described experimental conditions, which may indicate an efficient extraction of photoprotective constituents. Nevertheless, variations in solvent composition and experimental setup should be considered when interpreting quantitative differences across studies.

Regarding UVA protection (Figure S6b,c), all extracts were classified within the maximum UVAr category, showing strong UVA absorption, and their critical wavelengths (λ(crit)) corresponded to levels 3–4, the highest in the FDA classification [79,80]. These results suggest potential broad-spectrum behavior under the experimental conditions evaluated. However, it is important to note that these parameters were determined in solution and therefore represent an approximation of UVA absorption capacity; they do not replace standardized in vitro or in vivo assays performed on films or finished formulations required for regulatory broad-spectrum claims [81]. The observed absorption profiles are attributable to flavonoids and stilbenoids. As previously mentioned, flavonoids exhibit two characteristic absorption bands in the UV region, enabling coverage across both UVB (290–320 nm) and UVA (320–400 nm) regions, while stilbenoids, with maxima around 250–340 nm [82], also contribute to UVA absorption, consistent with the experimental findings.

## 3. Materials and Methods

### 3.1. Chemicals

The reagents used in this study were selected according to the analytical or bioactivity assays performed. AR-grade butanol, AR-grade ethanol, and n-hexane (PanReac AppliChem, ITW Reagents, Barcelona, Spain) were employed for extract preparation. Methanol-d_4_ (MeOD, 99.8%, Cambridge Isotope Laboratories, Tewksbury, MA, USA) and deuterium oxide (D_2_O, 99.9%, Cambridge Isotope Laboratories, Tewksbury, MA, USA) were used for NMR analysis.

For UHPLC-DAD-ELSD analysis, formic acid (reagent grade, Carlo Erba Reagents-SA, Sabadell, Spain), HPLC-grade acetonitrile, and deionized water (resistivity > 18 MΩ·cm), obtained from a Milli-Q Millipore^®^ system (Millipore Sigma, Burlington, MA, USA), were used as mobile phase components. LC-MS-grade water, acetonitrile, formic acid (100%), and ammonium formate (>99%) were employed for UHPLC-MS/MS chromatography.

For GC analysis, a boron trifluoride–methanol complex (BF_3_/MeOH) (Merck^®^, Merck, Darmstadt, Germany) was used for derivatization and Supelco^®^ 37 Component FAME Mix (Merck^®^) was used as standard mixture. Finally, spectrophotometric-grade ethanol (≥99.9%) (Merck^®^) and benzophenone-3 (Merck^®^) were used in the spectrophotometric assessment of photoprotective activity.

### 3.2. Plant Material and Extraction

The plant materials, including leaves, pericarps, and seeds, were obtained from *Passiflora ligularis*, *P. edulis* var. *edulis*, *P. edulis* var. *flavicarpa*, *P. quadrangularis*, *P. maliformis*, and the hybrid *P. tarminiana* × *P. tripartita*, sourced from commercial suppliers and local marketplaces (Table S4). The hybrid species was taxonomically identified by botanist Gustavo Morales based on morphological characteristics. All plant materials were shade-dried, stored at room temperature in the absence of light, ground, and sieved using a No. 20 mesh to ensure sample homogenization prior to extraction.

The butanolic fractions (BFs) from leaves and pericarps were obtained following the method described by Urrego [15]. Briefly, 20 g of dried and powdered material were subjected to infusion with distilled water (200 mL; 1:10 *w*/*v*) at 90 °C for 10 min. After filtration, the aqueous extract was partitioning with three sequential volumes of n-butanol (20 mL each). The organic phase was concentrated under reduced pressure and subsequently freeze-dried.

The hydroethanolic extract (HE) from seeds was obtained through ultrasound-assisted extraction, as described by Sepúlveda [24] and De Satana [21], with slight modifications. Briefly, 5 g od dried and powdered seeds were extractred with 50 mL of 70% ethanol (1:10 *v*/*w*) for a single 10 min cycle at 25 °C, using an Elmasonic EASY E30H (Elma^®^, Singen, Germany) ultrasonic bath (1.9 L capacity, 37 kHz frequency, 80 W power). The powdered seeds were extracted with The extracts were filtered, concentrated under reduced pressure, and freeze-dried. Extraction yields (%) were calculated gravimetrically based on dry weight.

Due to the high fatty acid content previously reported in Passiflora seeds [83], Fatty acid-rich hexane oils (FAHOs) were extracted in order to characterize their fatty acid composition. FAHOs were obtained from the seeds by dynamic maceration with n-hexane (1:5 *w*/*v*) for 1 h. The resulting extracts were filtered, and the solvent was evaporated under reduced pressure to obtain the extract as an oil.

### 3.3. NMR Preliminary Profiling

Proton nuclear magnetic resonance (^1^H NMR) spectra were recorded on a Bruker Advance II 400 MHz spectrometer. Methanol-d_4_ (CD_3_OD) was used as the solvent for the HE of seeds, while the BF from leaves and pericarps was analyzed using a deuterated solvent mixture (MD6), consisting of 0.5 mL of CD_3_OD and 0.5 mL of KH_2_PO_4_ buffer in D_2_O (pH 6.0), as previously applied in the analysis of *P. ligularis* leaves [23]. Spectra were acquired at 25 °C using 32 scans, relaxation delay of 1 s and the standard Bruker pulse sequence NOESYpr1d for water signal suppression. All spectra were processed using standard Fourier transformation procedures, including phase and baseline correction. For comparative purposes, defined spectral regions were integrated and the total integral area was normalized to 100 to allow relative comparison among species.

### 3.4. GC-FID and GC-MS Fatty Acids Analysis

FAHOs were subjected to a methylation reaction to obtain fatty acid methyl esters (FAMEs) following the method described by Araujo [84]. Specifically, 10 mg of FAHOs were mixed with 2 mL of BF_3_/CH_3_OH, heated to 100 °C for 1 h, and then cooled to room temperature. Subsequently, 1 mL of GC-grade hexane and 2 mL of deionized water were added, and the mixture was vortexed. The organic phase containing the FAMEs was separated and analyzed using gas chromatography.

The analysis was performed on an Agilent 8860 gas chromatograph equipped with a split/splitless injector and a flame ionization detector (FID). Samples were analyzed using an Agilent 19091s-436 column (60 m × 0.25 mm; 0.25 µm) under a 20 min temperature program: initial temperature of 100 °C, increasing to 300 °C at a rate of 10 °C/min. The injector and detector temperatures were set at 300 °C and 290 °C, respectively. A direct injection method was employed, with an injection volume of 1 µL and a split ratio of 1:100. Hydrogen was used as the carrier gas at a flow rate of 2 mL/min. Data acquisition and analysis were conducted using OpenLab Software (Version 2.8).

The identification of fatty acid methyl esters (FAMEs) present in the FAHOs samples was carried out by comparison with the Supelco^®^ 37 Component FAME Mix standard mixture. The relative abundances of the sample constituents are reported as an indicator of the proportional composition of the substances present in the sample.

The GC-MS analysis was performed using an Agilent 6890 gas chromatograph (Agilent Technologies, Santa Clara, CA, USA) coupled to a MSD 7790 mass selective detector, equipped with an SLB^®^5 ms Capillary column (30 m × 0.25 mm; 0.25 µm). The same temperature program described for the GC-FID analysis was applied.

### 3.5. UHPLC-DAD-ELSD Profiling

Samples were dissolved in ACN:H_2_O (1:1) at a concentration of 2 mg/mL and filtered through a 0.22 µm membrane prior to injection. The analysis was performed using a Dionex Ultimate 3000 (Thermo Fisher Scientific, Waltham, MA, USA) ultrahigh-performance liquid chromatograph, equipped with a Dionex Ultimate 3000RS quaternary pump, inline degasser, automatic injector, and coupled to a Dionex Ultimate 3000 diode array detector (DAD) and a Sedex 85 evaporative light scattering detector (ELSD) (80 °C, 40 psi). A Phenomenex^®^ (Phenomenex, Torrance, CA, USA) Kinetex C18 (100 mm × 2.1 mm; 2.6 µm) was used as the stationary phase, maintained at 40 °C, with a constant flow rate of 0.3 mL/min and an injection volume of 3 µL. The mobile phase consisted of a gradient system of 0.5% formic acid (Solvent A) and acetonitrile (Solvent B) with the following gradient program: 10–35%B (0–8 min), 35%B (8–9 min), 35–85% (9–15 min), 85%B (15–18 min), 85–10%B (18–22 min). DAD spectra were acquired from 200 nm to 800 nm. Data processing was carried out using Chromeleon client Software, version 6.8 SR15.

### 3.6. UHPLC-MS/MS Profiling

The UHPLC-MS/MS analysis was conducted using an UHPLC Ultimate 3000 (Thermo scientific) as previously describe, coupled to a Bruker Impact II UHR-q-TOF mass spectrometer (Bruker Daltonics, Bremen, Germany), equipped with electrospray ionization (ESI) source and the spectra were acquired in negative mode (BF of leaves, BF of pericarps, HE seeds) and positive mode (BF leaves). The samples were prepared at a concentration of 1 mg/mL and injected in triplicated. The same chromatographic system previous described was used. The mass spectrometer operated in data-dependent acquisition (DDA) mode. MS^1^ survey scans were acquired over a mass range of *m*/*z* 50–1500. Up to five MS/MS spectra per cycle were obtained in CID mode, selecting the five most intense precursor ions from each MS^1^ scan for fragmentation. Active exclusion was applied after two MS/MS spectra and released after 30 s to prevent repeated fragmentation of the same precursor ion. Precursors were reconsidered when the current intensity/previous intensity ratio reached 5.0 [85]. A ramped collision energy between 18 and 50 eV was employed for ion fragmentation, according to the isolation mass and charge state (Table S5). Ion source parameters are summarized in Table S6. Data were processed and converted to mzML format using Compass DataAnalysis software (version 4.3).

### 3.7. UHPLC-MS/MS Data Processing

The mzML files were pre-processed in Mzmine software, version 3.3.0., following the procedure suggested by Heuckeroth  [86]. The complete set of data-processing parameters applied in both MZmine and the GNPS FBMN workflow is provided in Supplementary Table S7.

For the FBMN generation, the resulting files (.mgf and _quant.csv) were uploaded, along with a metadata file, to the GNPS (https://gnps.ucsd.edu/ProteoSAFe/static/gnps-splash.jsp accessed on 19 March 2026) platform as part of their FBMN workflow. The samples were grouped according to the different parts of the plant evaluated (Leaves, pericarps and seeds) for network creation. The Feature-Based Networks can be visualized from the GNPS platform via the links provided in Table S8. These molecular networks were enhanced with the use of MolNetEnhacer tool, which provided chemical class annotations based on the Classyfire chemical ontology [87]. Access links are also shown in Table S8. The enhanced molecular networks were visualized and analyzed in Cytoscape software, version 3.10.2.

For SIRIUS annotation, the same pre-processing was carried out, and the resulting file (.mgf for SIRIUS) was uploaded to SIRIUS software, version 6.0.5., where CANOPUS and CSI:FingerID were applied.

### 3.8. Photoprotective Activity Assesment

Following the procedure described by Abril et al. [88], stock solutions of the polar extracts (BF and HE) (2 mg/mL) were prepared in spectroscopic-grade ethanol (≥99,9%) for the initial SPF screening. Serial dilutions (200, 100, 50, and 10 ppm) were prepared, and 200 µL of each was transferred to UV-transparent 96-well Corning™ plates. The absorbance of each sample was recorded in the 290–400 nm range, at 1 nm intervals, using a microplate reader (Varioskan™ LUX, Thermo Fisher Scientific Inc., Waltham, MA, USA). All measurements were performed in triplicate. Ethanol was used as the blank, and benzophenone-3 (BP-3) served as the positive control.

The sun protection factor (SPF) was calculated using the Mansur equation (Equation (1)) [89], where FC is a correction factor (10), Abs(λ) is the sample absorbance, EE(λ) the erythemal effect spectrum, and I(λ) the solar intensity spectrum, as defined in Table S9 [90].

(1) $$FPS=FC*\;\sum \begin{array}{c} 320\\ 290 \end{array}EE\left(\lambda \right)*I\left(\lambda \right)*Abs\left(\lambda \right)$$

The six extracts with the highest SPF in the initial screening were further analyzed using a cell spectrophotometer equipped with 1 cm quartz cuvettes over the 290–400 nm range. The same dilutions were evaluated in triplicate. SPF values were determined by the Mansur Equation (1), classifying samples as low (2–6), medium (8–12), high (15–25), very high (30–59), or ultra-high (≥ 50) [91].

It is important to note that this method provides a spectrophotometric in vitro screening based on solution absorbance and does not substitute for regulatory in vivo or in vitro SPF testing performed on finished formulations. The reported SPF classes should therefore be interpreted as comparative screening categories, as this approach does not account for formulation-dependent factors such as matrix interactions, spreadability, or film thickness.

The UVA ratio (UVAr) and critical wavelength (λ_crit_) were determined following reported methods [88,91]. UVAr (Equation (2)) compares absorbance in the UVA and UVB regions, allowing classification as very low (0.0–0.2), moderate (0.2–0.4), good (0.4–0.6), superior (0.6–0.8), or maximum (≥0.8) [92]. The λ_crit_ (Equation (3)) corresponds to the wavelength at which 90% of the integrated absorbance (290–400 nm) is reached, indicating UVA coverage. According to FDA criteria, samples are rated from level 0 (<325 nm) to level 4 (≥370 nm) [91].

(2) $$UVAr=\frac{\int_{320}^{400}Abs\left(\lambda \right)d\lambda }{\int_{290}^{320}Abs\left(\lambda \right)d\lambda }$$

(3) $$\int_{290}^{\lambda crit}Abs\left(\lambda \right)d\lambda =0.9\times \int_{290}^{400}Abs\left(\lambda \right)d\lambda$$

### 3.9. Statistical Analysis

Statistical differences among samples in the photoprotection assays were evaluated using one-way analysis of variance (ANOVA). When significant differences were detected, Tukey’s test was applied for multiple comparisons at a significance level of *p* < 0.05. Statistical analyses were carried out using R (RStudio environment, Version 2026.01.1+403).

## 4. Conclusions

Preliminary ^1^H NMR profiling and chromatographic analyses revealed clear compositional differences among *Passiflora* byproducts. Leaf BFs of the six evaluated *Passiflora* species were characterized by a high diversity of flavonoids and saponins, whereas pericarp BFs were predominantly enriched in sugar-type compounds. Seed HEs were mainly composed of fatty acids and stilbenoids, with linoleic acid as the major fatty acid across species. *P. quadrangularis* seeds exhibited a distinct fatty acid profile, including myristic acid and an additional unsaturated fatty acid.

UHPLC–MS/MS analysis combined with GNPS-based molecular networking enabled the dereplication of 52 compounds in leaf and pericarp BFs, including 31 flavonoids and 16 saponins, confirming flavonoids as the predominant chemical class. In contrast, seed HEs yielded 22 annotated compounds and exhibited a generally consistent stilbenoid-rich profile across species, with piceatannol, trans-resveratrol, and scircupsin B being the most abundant constituents. An exception was *P. tarminiana* × *P. tripartita*, which lacked stilbenoids and instead showed a predominance of chalcone-type compounds. Despite this overall consistency, seed extracts also displayed diversity in minor constituents, including species-specific flavonoids.

These compositional differences were reflected in the photoprotective screening results. Leaf BFs, particularly from *P. edulis* var. *flavicarpa* (SPF_200_ ppm = 23.27 ± 0.79) and *P. maliformis* (SPF_200_ ppm = 21.89 ± 0.22) exhibited the highest SPF values under the evaluated conditions, along with maximum UVAr classification and critical wavelength (λ_crit_) values corresponding to levels 3–4. Seed HEs showed moderate to high SPF values, notably in *P. ligularis* (SPF_200_ ppm = 18.96 ± 0.03) and *P. maliformis* (SPF_200_ ppm = 15.51 ± 0.17), consistent with their stilbenoid content. These findings support the contribution of flavonoids and stilbenoids to UV absorption in both the UVB and UVA regions. However, it should be emphasized that SPF, UVAr, and λ_crit_ were determined in solution as spectrophotometric screening parameters and do not replace standardized in vitro or in vivo assays performed on sunscreen films or finished formulations. Furthermore, photostability and toxicological evaluations were not addressed and remain necessary for future product development.

Overall, this integrative chemical profiling approach highlights the value of combining complementary analytical platforms for compound annotation in complex botanical matrices. The results support the potential valorization of *Passiflora* agro-industrial byproducts as promising sources of UV-absorbing compounds, while recognizing the need for further formulation-based and safety studies to substantiate cosmetic applications.

## Figures and Tables

**Table 1:** Relative abundance of Fatty acids identified in the FAHOs evaluated *Passiflora* seeds, reported as methyl ester form (FAMEs).

Species	Methyl Myristate G1	Methyl Palmitoleate ^1^ G2	Methyl Palmitate G3	Methyl Linoleate G4	Methyl Oleate G5	Methyl Stearate G6
*P. ligularis*	-	-	8.3	74.6	15.2	1.9
*P. edulis* var. *edulis*	-	-	7.7	79.5	10.9	1.9
*P. edulis* var. *flavicarpa*	-	-	8.8	75.2	14.1	1.9
*P. maliformis*	-	-	10.1	69.9	17.6	2.4
*P. quadrangularis*	1.2	1.5	14.9	57.1	22.5	2.8
*P. tarminiana* × *P. tripartita*	-	-	9.8	74.2	13.3	2.7

^1^ Proposed by GC-MS analysis.

**Table 2:** Principal Compounds Identified in BFs of *Passiflora* Leaves and Pericarps.

Peak	Compound Name	Experimental [M − H]^−^	RT (min) UHPLC-MS/MS ^1^	Principal Fragments (*m*/*z*)	UV λ Max (nm)	Dereplication Tool ^2^	Compound Classification	CF_Class/CF_Dparent ^3^	CANOPUS (Most Specific Class) ^4^	Molecular Formula (Error in ppm)	COSINE (GNPS) ^5^	CSI:FingerIDScore ^6^	Species-Part of the Plant ^7^	Dereplication Level ^8^
1	-	340.1043	3.22	340; 294; 188; 161	-	GNPS/SIRIUS	Glycosylated compound	Organooxygen Compounds/N-acyl-alpha-hexosamines	Cyanogenic glycosides	-	-	-	*P. edulis* var. *flavicarpa* (P); *P. edulis* var. *edulis* (P)	Level 3
2	Saponarin	593.1518	3.84	593; 431; 311; 297; 282	335; 270; 226	GNPS/SIRIUS	Diglycosylated flavonoid	Flavonoids/Flavonoids-7-*O*-glycosides	Flavonoid-7-*O*-glycosides	1.97	0.95	−12.942	*P. maliformis* (L)	Level 2
3	Shaftoside/Corymboside	563.141	3.97	563; 383; 353	340; 270; 226	GNPS/SIRIUS	Flavonoid	Flavonoids/Flavonoids-3-*O*-glycosides	Flavonoid-3-*O*-glycosides	1.67	-	−2.775	*P. maliformis* (P)	Level 2
4	Isoorientin	447.0927	4.06	447; 357; 339; 327; 297; 285	349; 269; 225	Literature [1,45]/GNPS/SIRIUS	Glycosylated flavonoid	Flavonoids/Flavonoids-7-*O*-glycosides	Flavonoid-8-*C*-glycosides	0	0.94	−10. 339	*P. edulis* var. *flavicarpa* (L,P)	Level 2
5	Apigenin-6-*C*-arabinosyl-7-*O*-glucoside	563.1406	4.20	563; 473; 401; 341; 311; 297; 282	338; 270; 222	Literature [26]/GNPS/SIRIUS	Glycosylated flavonoid	Flavonoids/Flavonoids-7-*O*-glycosides	Flavonoid-7-*O*-glycosides	0.95	-	-	*P. maliformis* (L)	Level 2
6	6-[4,5-dihydroxy-6-(hydroxymethyl)-3-[(3,4,5-trihydroxy-6-methyloxan-2-yl)oxy]oxan-2-yl]-5,7-dihydroxy-2-(4-hydroxyphenyl)-4H-chromen-4-one	577.1667	4.27	577; 487; 457; 367; 337; 309; 281	310; 273; 223	GNPS/SIRIUS	Glycosylated flavonoid	Flavonoids/Flavonoids-7-*O*-glycosides	Flavonoid 8-*C*-glycosides	-	-	−28.796	*P. edulis* var. *flavicarpa* (L,P)	Level 2
7	vitexin-2″-*O*-glucoside	593.1525	4.44	593; 413; 293	335; 268; 226	Literature [2]/GNPS/SIRIUS	Glycosylated flavonoid	Flavonoids/Flavonoids-7-*O*-glycosides	Flavonoid-7-*O*-glycosides	3.15	-	-	*P. quadrangularis* (L,P)	Level 2
8	vitexin-2″-*O*-xyloside	563.1401	4.64	563; 413; 311; 293	335; 268; 226	Literature [2]/GNPS/SIRIUS	Glycosylated flavonoid	Flavonoids/Flavonoids-7-*O*-glycosides	Flavonoid-7-*O*-glycosides	0.16	-	-	*P. quadrangularis* (L,P)	Level 2
9	luteolin-4-*O*-glucopyranosyl, 8-*C*-(6″ acetyl)-glucopyranoside	651.1559	4.88	651; 607; 489; 357; 327	335; 269; 223	Literature [38]/GNPS/SIRIUS	Glycosylated flavonoid	Flavonoids/Flavonoids-3-*O*-glycosides	Flavonoid-7-*O*-glycosides	1.23	-	-	*P. tarminiana* × *P. tripartita* (P)	Level 3
10	Vitexin	431.0988	4.88	431; 341; 311; 283	327; 270; 226	Literature [1,45]/GNPS/SIRIUS	Glycosylated flavonoid	Flavonoids/Flavonoids-7-*O*-glycosides	Flavonoid-8-*C*-glycosides	0.93	-	−47.93	*P. edulis* var. *flavicarpa* (L); *P. maliformis* (L)	Level 2
11	-	637.1755	4.94	637; 475; 457; 439; 379; 337; 313; 295; 283	327; 270; 226	GNPS/SIRIUS	Glycosylated flavonoid	Flavonoids/Flavonoids-*O*-glucuronides	Flavonoid-8-*C*-glycosides	2.2	-	-	*P. tarminiana* × *P. tripartita* (L)	Level 3
12	Quercetin 3-*O*-glucoside	463.0885	5.05	463; 301; 300; 271; 255; 151	352; 256; 229	Literature [23,24]/GNPS	Flavonoid 3-*O*-glycosylated	Flavonoids/Flavonoids-3-*O*-glycosides	Flavonoid-3-*O*-glycosides	1.84	0.99	-	*P. ligularis* (L)	Level 2
13	-	797.2137	5.06	797; 635; 471; 327	331; 269; 223	GNPS/SIRIUS	Glycosylated flavonoid	Flavonoids/Flavonoids-3-*O*-glycosides	Flavonoid-3-*O*-glycosides	-	-	-	*P. maliformis* (P)	Level 3
14	Isovitexin	431.0979	5.12	431; 341; 311; 283	336; 270; 226	Literature [1,45]/GNPS/SIRIUS	Glycosylated flavonoid	Flavonoids/Flavonoids-7-*O*-glycosides	Flavonoid-8-*C*-glycosides	0.23	0.92	−11.007	*P. edulis* var. *flavicarpa* (L,P)	Level 2
15	-	417.0821	5.13	417; 357; 327; 298; 285	340; 270; 227	GNPS/SIRIUS	Glycosylated flavonoid	Flavonoids/Flavonoids-7-*O*-glycosides	Flavonoid-8-*C*-glycosides	-	-	-	*P. maliformis* (L)	Level 3
16	4′-methoxyluteolin-6-*C*- glucopyranoside	461.1083	5.24	461; 371; 341; 298	346; 268; 227	Literature [38]/SIRIUS	Glycosylated flavonoid	-	Flavonoid *C*-glycoside	1.3	-	-	*P. tarminiana* × *P. tripartita* (L)	Level 2
17	4′-methoxyluteolin-8-*C*-glucopyranoside	461.1083	5.38	461; 371; 341; 298	345; 269; 226	Literature [38]	Glycosylated flavonoid	-	-	1.3	-	-	*P. tarminiana* × *P. tripartita* (L)	Level 2
18	-	455.2131	5.42	455; 409; 263; 161	353; 256; 229	GNPS	Glycosylated compound	Organooxygen Compounds/*O*-glycosyl compounds	Alkyl glycosides	-	-	-	*P. edulis* var. *edulis* (P)	Level 3
19	Quercetin 3-*O*-malonylglucoside	549.0880	5.47	505; 301;300; 271; 178; 151	353; 256: 229	Literature [23,24]/GNPS/SIRIUS	Flavonoid 3-*O*-glycosylated	Flavonoids/Flavonoids-3-*O*-glycosides	Flavonoid-3-*O*-glycosides	0.07	0.91	−6.973	*P. ligularis* (L)	Level 2
20	Kaemferol 3-*O*-glucoside	447.0330	5.76	447; 327; 307; 284; 255; 227	350; 256	Literature [23,24]/GNPS	Flavonoid 3-*O* glycosylated	Flavonoids/Flavonoids-3-*O*-glycosides	Flavonoid-3-*O*-glycosides	132	0.99	-	*P. ligularis* (L,P)	Level 2
21	-	433.1125 (+) *	5.76	433; 415; 397; 379; 353; 329;299	348; 250;245; 226	-	Glycosylated compound	Flavonoids/Flavonoids-*O*-glucuronides	Phenolic glycoside	-	-		*P. edulis* var. *flavicarpa* (L,P); *P. edulis* var. *edulis* (P)	Level 3
22	-	417.2126	5.86	417; 371; 327	341;255; 216	GNPS	Glycosylated compound	Flavonoids/Flavonoids-3-*O*-glycosides	Terpene glycosides	-	-	-	*P. maliformis* (P)	Level 3
23	Apigenin 7-*O*-glucoside	431.0988	5.97	431; 288; 240; 211; 151	335; 266; 228	Literature [23,24]/GNPS/SIRIUS	Flavonoid 7-*O*-glycosylated	Flavonoids/Flavonoids-3-*O*-glycosides	Flavonoid-7-*O*-glycosides	2.60	0.97	-	*P. ligularis* (L)	Level 2
24	Luteolin-8-*C*-(6″ acetyl)- glucopyranoside	489.1031	5.97	489;357; 327; 299; 297	347; 256; 226	Literature [38]/GNPS/SIRIUS	Glycosylated flavonoid	Flavonoids/flavonoid-3-*O*-glycosides	Flavonoid-3-*O*-glycosides	1.64	-	-	*P. tarminiana* × *P. tripartita* (L,P)	Level 2
25	4′-methoxyluteolin-6-*C*-6″acetylglucopyranoside	503.1183	6.19	503; 443; 428; 383; 241; 298	346; 265; 226	Literature [38]	Glycosylated flavonoid	-	-	2.39	-	-	*P. tarminiana* × *P. tripartita* (L,P)	Level 2
26	-	401.087	6.2	401; 341; 311; 283; 282	336; 270; 223	GNPS	Flavonoid	Flavonoids/Flavonoids-7-*O*-glycosides	Flavonoid *C*-glycosides	-	-	-	*P. maliformis* (L)	Level 3
27	-	469.2286	6.27	469; 423; 277; 161	348; 256; 229	GNPS	Glycosylated compound	Organooxygen Compounds/*O*-glycosyl compounds	Alkyl glycosides	-	-	-	*P. edulis* var. *edulis* (P)	Level 3
28	Kaemferol-3-*O*-(6″acetil) glucoside	489.1037	6.31	489; 284; 255; 227	346; 254; 228	Literature [23,24]/GNPS/SIRIUS	Flavonoid 3-*O*-glycosylated	Flavonoids/Flavonoids-3-*O*-glycosides	Flavonoid-3-*O*-glycosides	6.44	-	-	*P. ligularis* (L,P)	Level 2
29	Kaemferol- 3-(6″-malonylglucoside)	533.0927	6.32	489; 448; 403; 285; 255; 227	346; 265; 229	Literature [23,24]/GNPS/SIRIUS	Flavonoid 3-*O*-glycosylated	Flavonoids/Flavonoids-3-*O*-glycosides	Flavonoid-3-*O*-glycosides	0.53	-	-	*P. ligularis* (L)	Level 2
30	-	433.113 (+) *	6.52	433; 415; 397; 379; 353; 329; 299	347; 250;245; 226	GNPS/SIRIUS	Glycosylated flavonoid	Flavonoids/Flavonoids-*O*-glucuronides	*C*-glycosyl compounds	-	-		*P. edulis* var. *flavicarpa* (L,P)	Level 4
31	-	575.1409	6.58	575; 411; 301; 297; 285	348; 256; 224	SIRIUS	Glycosylated flavonoid	-	Flavonoid-8-*C*-glycosides	-	-	-	*P. edulis* var. *edulis* (L,P)	Level 2
32	Isoramnetin 3-*O*-(6″–malonyl) glucoside	565.1164 (+) *	6.6	317; 302; 285; 231; 159	352; 255; 228	Literature [23,24]/GNPS/SIRIUS	Flavonoid 3-*O*-glycosylated	Carboxilic acids and derivatives/Proline and derivatives	Flavonoid-3-*O*-glycosides	5.48	-	−18.349	*P. ligularis* (L)	Level 2
33	Apigenin-8-*C* (6″ acetyl)- glucopyranoside	473.1087	6.61	473; 413; 311; 283	335; 268; 226	Literature [38]	Glycosylated flavonoid	-	-	0.42	-	-	*P. tarminiana* × *P. tripartita* (L)	Level 2
34	4′-methoxyluteolin-6-*C*- 8″acetylglucopyranoside	503.1193	6.94	503; 341; 298	345; 254; 227	Literature [38]	Glycosylated flavonoid	-	-	0.4	-	-	*P. tarminiana* × *P. tripartita* (L,P)	Level 2
35	Apigenin	269.0459	7.04	269; 201; 159; 117	335; 266; 229	Literature [23,24]/GNPS/SIRIUS	Flavone	Flavonoids/Flavones	Chromones	3.87	0.94	−6.703	*P. ligularis* (L,P)	Level 2
36	-	561.1610	7.49	561; 253; 179	305; 267; 222	-	Glycosylated compound	-	-	-	-	-	*P. edulis* var. *edulis* (L)	Level 2
37	-	469.1709	7.56	469; 367; 325; 243; 203; 163; 148; 125	409; 312; 214	GNPS	Glycosylated flavonoid	Flavonoids/Flavonoids-3-*O*-glycosides	Saccharolipids	-	-	-	*P. ligularis* (P)	Level 3
38	PR309185	461.1092	7.79	415; 293; 253; 237; 209	305; 267; 228	GNPS	Flavonoid 7-*O*-glycosylated	Flavonoids/Flavonoids-3-*O*-glycosides	Flavonoid-7-*O*-glycosides	1.73	0.9	-	*P. ligularis* (L,P)	Level 2
39	Ciclopassifloside IX	861.4849(+) *	8.21	861; 681; 645; 501; 483; 465; 447; 437; 419	-	Literature [15]	Saponin	-	-	0.12	-	-	*P. edulis* var. *edulis* (L)	Level 3
40	Chrysin	253.0492	8.79	243; 209; 243; 119; 107	304; 267	Literature [23,24]/GNPS/SIRIUS	Flavone	Flavonoids/Flavones	Isoflavones	3.47	-	−5.75	*P. ligularis* (L,P)	Level 2
41	Ciclopassifloside XI	861.4856 (+) *	9.09	861; 681; 645; 501; 483; 465; 447; 419	-	Literature [15]	Saponin	-	-	0.7	-	-	*P. edulis* var. *edulis* (L)	Level 3
42	-	943.5274 (+) *	9.52	601; 439; 421; 403; 383	-	GNPS/SIRIUS	Saponin	Steroids and steroids derivatives/Steroidal glycosides	Curcubitacin glycosides	-	-	-	*P. quadrangularis* (L)	Level 3
43	Ciclopassifloside III	867.4672 (+) *	9.91	867; 827; 665; 499; 485; 467; 343	-	Literature [15]	Saponin	-	-	5.53	-	-	*P. edulis* var. *edulis* (L)	Level 3
44	-	1105.5797 (+) *	9.92	943; 781; 601; 439; 421; 325; 163	-	GNPS/SIRIUS	Saponin	Steroids and steroids derivatives/Steroidal glycosides	Curcubitacin glycosides	-	-	-	*P. quadrangularis* (L)	Level 3
45	1-α- hidroxi-quadranguloside A/1-β-hidroxi-quadranguloside B	983.5229 (+) *	10.54	983; 857; 648; 550; 439	-	Literature [63]	Saponin	-	-	0	-	-	*P. quadrangularis* (L)	Level 3
46	1-β-Quadranguloside C	821.4734 (+) *	11.22	821; 602; 422	-	Literature [63]	Saponin	-	-	8.64	-	-	*P. quadrangularis* (L)	Level 3
47	Quadranguloside B	945.5453 (+) *	11.43	765; 603; 423; 325	-	Literature [63]/GNPS/SIRIUS	Saponin	Steroids and steroids derivatives/Steroidal glycosides	Curcubitacin glycosides	3.17	-	-	*P. quadrangularis* (L)	Level 3
48	Ligularoside C	850.4946 (+) ^*^	11.72	366; 204	-	Literature [23,24]/GNPS/SIRIUS	Saponin	Glycerophospholipids/Macrolide lactams	Triterpene saponins	1.02	-	-	*P. ligularis* (L)	Level 3
49	-	936.4938 (+) *	12.01	452; 204; 168; 138	-	-	Saponin	-	-	-	-	-	*P. ligularis* (L)	Level 4
50	3-*O*-β-D-glycopyranosil-(1-2)-β-D-glucopyranoside of oleanolic acid	781.3473 (+) *	12.08	783; 619; 601; 439; 421	-	Literature [63]/GNPS/SIRIUS	Saponin	Steroids and steroids derivatives/Steroidal glycosides	Triterpene saponins	87.66	-	-	*P. quadrangularis* (L)	Level 3
51	Quadranguloside C	783.4889 (+) *	12.56	604; 441; 423	-	Literature [63]/GNPS/SIRIUS	Saponin	Steroids and steroids derivatives/Steroidal glycosides	Triterpene saponins	0	-	-	*P. quadrangularis* (L)	Level 3
52	Ligularoside B	834.4992 (+) *	13.49	366; 204; 138	-	Literature [23,24]/GNPS/SIRIUS	Saponin	Glycerophospholipids/Macrolide lactams	Triterpene saponins	1.52	-	-	*P. ligularis* (L)	Level 3

* Pseudomolecular ion detected in positive ionization mode [M + H]. ^1^ UHPLC-MS/MS retention time. ^2^ Source of dereplication information used (identification, chemical classification). ^3^ Chemical classification given by MolNetEnhacer (GNPS) in two levels, class and parent. ^4^ Chemical classification given by CANOPUS (SIRIUS). This was taken at the most specific level determined. ^5^ Similarity factor generated by GNPS, ranging from 0 to 1, where values close to 1 indicate a higher similarity between the fragmentation patterns and the compound reported in the literature. ^6^ Similarity score generated by SIRIUS, for which values closer to 0 indicate a better match between the fragmentation patterns. ^7^ L: Leaves; P: Pericarps. ^8^ Dereplication level based on the criteria established by [64,65].

**Table 3:** Principal Compounds Identified in HEs of *Passiflora* seeds.

Peak	Compound Name	Experimental [M − H]^−^	RT (min) UHPLC-MS/MS ^1^	Principal Fragments (*m*/*z*)	UV λ Max (nm)	Dereplication Tool ^2^	Compound Classification	CF_Class/CF_Dparent ^3^	CANOPUS (Most Specific Class) ^4^	Molecular Formula (Error in ppm)	COSINE (GNPS) ^5^	CSI:FingerIDScore ^6^	Species	Dereplication Level ^7^
S1	Catechin	289.0717	2.98	289; 245; 203; 137; 125; 109	350; 281; 226	GNPS/SIRIUS	Hydroxychalcone	Linear 1,3-diarylpropanoids/2′-Hydroxychalcones	Catechins	1.73	0.96	−8.35	*P. tarminiana* × *P. tripartita*	Level 2
S2	Piceatannol	243.0668	5.27	225; 201; 175; 159	322; 228; 193	Literature [22,48]/GNPS/SIRIUS	Stilbenoid	Psoralens/Coumarins and der	Stilbenes	4.65	0.88	−3.97	*P. edulis* var. *flavicarpa*; *P. edulis* var. *edulis;* *P. maliformis*; *P. ligularis*; *P. quadrangularis*	Level 2
S3	-	435.1297	5.92	389; 257; 241	320; 230	GNPS/SIRIUS	Flavonoid	Flavonoids/2-arylbenzofuran flavonoids	Stilbene glycosides	-	-	-	*P. maliformis*; *P. quadrangularis*	Level 3
S4	-	433.1394	6.28	433; 403; 329	350; 220	SIRIUS	-	-	Tetrahydrofuran lignans	-	-	-	*P. ligularis*	Level 3
S5	-	271.0978	6.42	271; 227; 143; 115	350; 213; 205	SIRIUS	Stilbenoid	-	Stilbenes	-	-	-	*P. edulis* var. *flavicarpa*	Level 3
S6	-	299.0557	6.45	299; 284; 255; 227; 148	350; 310; 218	GNPS/SIRIUS	-	-	3-*O*-methylated flavonoids	-	-	-	*P. tarminiana* × *P. tripartita*	Level 3
S7	Trans-Resveratrol	227.0714	6.73	227; 185; 143	306; 193	Literature [22,48]/GNPS/SIRIUS	Stilbenoid	Psoralens/Coumarins and der	Stilbenes	2.64	0.94	−3.48	*P. edulis* var. *flavicarpa* *P. edulis* var. *edulis* *P. maliformis* *P. ligularis*	Level 2
S8	-	283.0248	6.97	283; 255; 227; 161; 134	390; 370; 310; 227	GNPS/SIRIUS	Flavonoid	Isoflavonoid/Coumestan	Xanthones	-	-	-	*P. tarminiana* × *P. tripartita*	Level 3
S9	Cyperusphenol B	727.1830	7.08	727; 617; 495; 375; 241	386; 281; 191	Literature [48,69]	Stilbenoid	-	3-hydroxyflavonoids	1.93	-	-	*P. edulis* var. *flavicarpa*	Level 2
S10	Scircupsin B	485.1241	7.17	485; 375; 243; 109	328;259; 207	Literature [48,69]	Stilbenoid	-	3′-hydroxyflavonoids	1.03	-	-	*P. edulis* var. *flavicarpa* *P. edulis* var. *edulis* *P. maliformis* *P. ligularis*	Level 2
S11	-	677.1875	7.23	631; 499; 241	326; 229	GNPS/SIRIUS	Flavonoid	Flavonoids/2-arylbenzofuran flavonoids	2-arylbenzofuran flavonoids	-	-	-	*P. quadrangularis*	Level 3
S12	7,3′,4′-Trihydroxyflavone	269.0453	7.33	269; 213; 133	393; 258;210	GNPS/SIRIUS	Flavone	Linear 1,3-diarylpropanoids/2′-Hydroxychalcones	Primary alcohols	1.23	0.89	-	*P. edulis* var. *edulis*	Level 2
S13	Isorhapontigenin	257.0816	7.52	257; 241; 213	316; 292; 234	GNPS/SIRIUS	Stilbenoid	Coumarins and d/Benzoxepines	Stilbenes	0.89	0.89	−30.25	*P. quadrangularis*	Level 2
S14	Cyperusphenol D	725.1641	7.52	725; 617; 493; 361; 240	325; 260; 212	Literature [48]	Stilbenoid	-	Benzoic acids and derivatives	2.48	-	-	*P. maliformis*	Level 2
S15	-	493.2277	7.58	493; 447; 101	328; 300; 228	-	-	-	-	-	-	-	*P. edulis* var. *edulis*	Level 4
S16	-	507.2427	7.81	507; 461; 315	330; 309; 223	-	-	-	-	-	-	-	*P. edulis* var. *edulis*	Level 4
S17	-	515.1340	8.07	469; 385; 241	324; 228	GNPS/SIRIUS	Flavonoid	2-arylbenzofuran flavonoids/Flavonoids	oligomeric stibenes	-	-	-	*P. maliformis*; *P. edulis* var. *flavicarpa*	Level 3
S18	-	271.0578	8.15	271; 253; 227; 209	330; 228; 214	SIRIUS	-	-	Resorcinols	-	-	-	*P. maliformis*	Level 3
S19	-	773.1795	8.24	727; 617; 495	332; 229; 201	-	-	-	-	-	-	-	*P. maliformis*; *P. ligularis*	Level 4
S20	-	269.0817	8.35	269; 253; 225	346; 230; 212	SIRIUS	-	-	2′hydroxychalcones	1.23	-	-	*P. tarminiana* × *P. tripartita*	Level 3
S21	Narigerin	271.0608	8.71	271; 253; 227; 209; 143	380; 217	GNPS/SIRIUS	Hydroxychalcone	Linear 1,3-diarylpropanoids/2′-Hydroxychalcones	2′hydroxychalcones	0.85	-	−42.34	*P. edulis* var. *edulis*; *P. tarminiana* × *P. tripartita*	Level 2
S22	Isoliquiritigenin	255.0658	10.25	255; 135; 119	367; 237; 212	GNPS/SIRIUS	Hydroxychalcone	Linear 1,3-diarylpropanoids/2′-Hydroxychalcones	4′-hydroxychalcones	0.51	0.95	−19.05	*P. edulis* var. *edulis*; *P. tarminiana* × *P. tripartita*	Level 2

Pseudomolecular ion detected in positive ionization mode [M + H]. ^1^ UHPLC-MS/MS retention time. ^2^ Source of dereplication information used (identification, chemical classification). ^3^ Chemical classification given by MolNetEnhacer (GNPS) in two levels, class and parent. ^4^ Chemical classification given by CANOPUS (SIRIUS). This was taken at the most specific level determined. ^5^ Similarity factor generated by GNPS, ranging from 0 to 1, where values close to 1 indicate a higher similarity between the fragmentation patterns and the compound reported in the literature. ^6^ Similarity score generated by SIRIUS, for which values closer to 0 indicate a better match between the fragmentation patterns. ^7^ Dereplication level based on the criteria stablished by [64,65].

## Data Availability

All relevant data supporting the findings of this study, including raw datasets, will be made freely available to any researcher for non-commercial purposes upon reasonable request, without breaching participant confidentiality.

## Supplementary Materials

The following supporting information can be downloaded at https://www.mdpi.com/article/10.3390/plants15060972/s1: Figure S1: ^1^H NMR spectra of the evaluated Passiflora Butanolic fractions (BFs) from pericarps in MD6; Figure S2: ^1^H NMR spectra of the evaluated Passiflora Hydroethanolic Extracts (HEs) from seeds in MeOD; Figure S3: GC-FID chromatograms of fatty acid methyl esters (FAMEs) from Passiflora seed FAHOs; Figure S4: Peak G2 MS/MS fragmentation; Figure S5: Most representative clusters of the molecular networks for leaf butanolic fractions; Figure S6: Most representative clusters of the molecular networks for pericarps BFs in negative ionization mode; Figure S7: Most representative clusters of the molecular networks for seeds HEs in negative ionization mode; Figure S8: Sun protection factor (SPF) screening; Figure S9: Photoprotection determined by spectrophotometry for the selected extracts: Table S1: Extraction yields of BFs and HEs. Table S2: Fatty acids identified in the FAHOs from the evaluated Passiflora species, reported as methyl ester form (FAMEs); Table S3: Screening SPF values at 100 ppm and Tukey’s multiple comparison test among samples; Table S4: Collection data (location and date) of the commercial Passiflora species studied; Table S5: Collision energy ramp applied for ion fragmentation in MS/MS analysis; Table S6: Ion source parameters for UHPLC-MS/MS analysis; Table S7: Data processing parameters for UHPLC-MS/MS analysis using MZmine and GNPS; Table S8: Access links to workflows performed in GNPS for each analyzed group; Table S9: EE(λ) × I(λ) value established for each evaluated wavelength.

## Abbreviations

The following abbreviations are used in this manuscript:

^1^H-NMR	Proton nuclear magnetic resonance
ACN	Acetonitrile
ANOVA	Analysis of variance
BF	Butanolic fraction
CF	ClassyFire
DAD	Diode array detector
DDA	Data-dependent acquisition
Dparent	Direct parent
ESI	Electrospray Ionization
ELSD	Evaporative light scattering detector
FAHO	Fatty acid-rich hexane oil
FAME	Fatty acid methyl esters
FBMN	Feature-based molecular networking
GC-FID	Gas chromatography–flame ionization detection
GC-MS	Gas chromatography–mass spectrometry
GNPS	Global Natural Products Social Molecular Networking
HE	Hydroethanolic extract
HRMS	High-Resolution Mass Spectrometry
LC-MS/MS	Liquid chromatography–tandem mass spectrometry
LC-PDA	Liquid chromatography–photodiode array detection
SPF	Sun Protection Factor
UHPLC	Ultra-high-performance liquid chromatography
UHR-q-TOF	Ultra-High Resolution Quadrupole Time-of-Flight
UVAr	UVA ratio
λ_crit_	Critical wavelength
