# Supplementary material for: Chemical Profiling and Photoprotective Activity of Extracts from Colombian Passiflora Byproducts
Source: Plants (Basel). 2026 Mar 21;15(6):972. doi: 10.3390/plants15060972 (PMC13030438; doi:10.3390/plants15060972)
Supplement: Supplementary file 1 [file plants-15-00972-s001.zip › plants-4169258-supplementary.pdf]

Article

Chemical Profiling and Photoprotective Activity of Extracts from Colombian Passiflora Byproducts

María Cabeza [1], Cindy Lucero López [1], Geison Modesti Costa [2,*], Mónica Ávila-Murillo [3], Freddy A. Ramos [3], Yolima Baena [1], Marcela Aragón Novoa [1] and Leonardo Castellanos [3]

1 Departamento de Farmacia, Facultad de Ciencias, Universidad Nacional de Colombia, Sede Bogotá, Carrera 30 # 45-03, Bogotá 11001, Colombia; mcabezap@unal.edu.co (M.C.); cillopezra@unal.edu.co (C.L.L.); ybaenaa@unal.edu.co (Y.B.); dmaragonn@unal.edu.co (M.A.N.)

2 Departamento de Química, Facultad de Ciencias, Pontificia Universidad Javeriana, Sede Bogotá, Carrera 7 # 40-62, Bogotá 110231, Colombia

3 Departamento de Química, Facultad de Ciencias, Universidad Nacional de Colombia, Sede Bogotá, Carrera 30 # 45-03, Bogotá 11001, Colombia; mcavilam@unal.edu.co (M.Á.-M.); faramosr@unal.edu.co (F.A.R.); lcastellanosh@unal.edu.co (L.C.)

* Correspondence: modesticosta.g@javeriana.edu.co

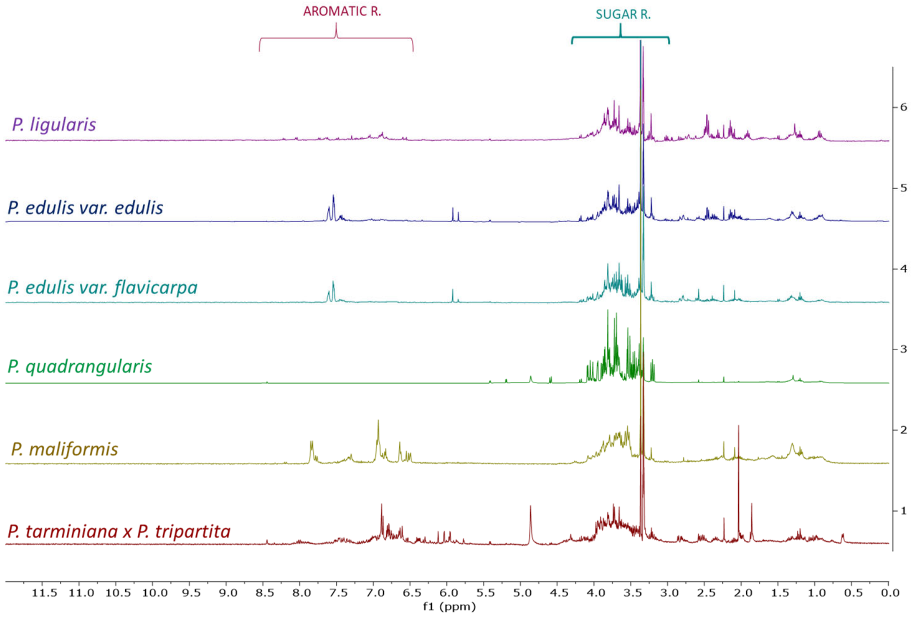

Figure S1: ^{1}H NMR spectra of the evaluated *Passiflora* Butanolic fractions (BFs) from pericarps in MD6

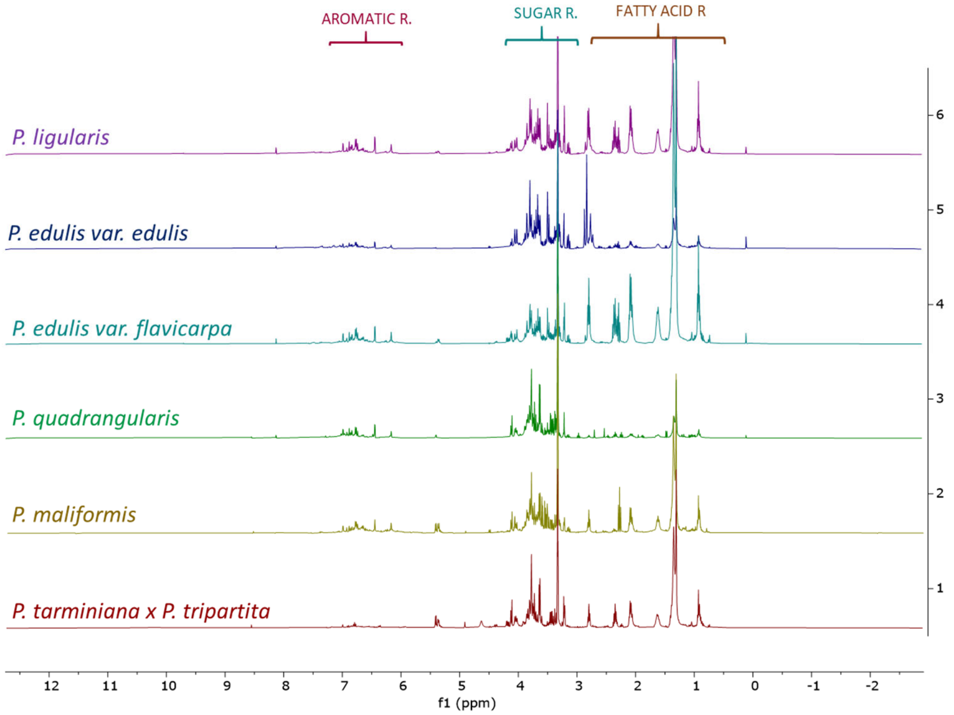

Figure S2: ^{1}H NMR spectra of the evaluated *Passiflora* HEs from seeds in MeOD.

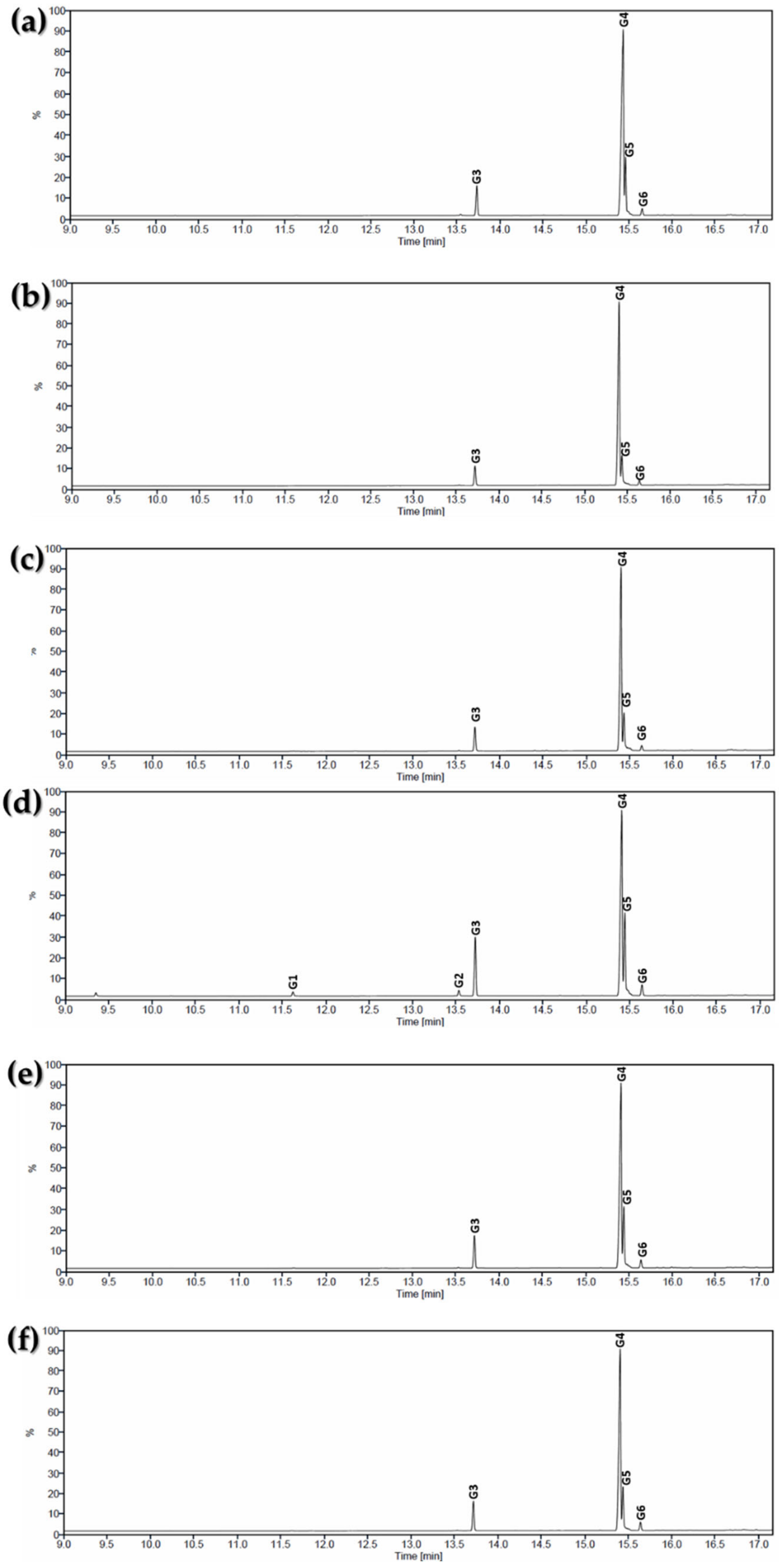
(a)
G3
G4
G5
G6
%
Time [min]
(b)
G3
G4
G5
G6
%
Time [min]
(c)
G3
G4
G5
G6
%
Time [min]
(d)
G1
G2
G3
G4
G5
G6
%
Time [min]
(e)
G3
G4
G5
G6
%
Time [min]
(f)
G3
G4
G5
G6
%
Time [min]

Figure S3: GC-FID chromatograms of fatty acid methyl esters (FAMEs) from Passiflora seed FAHOs: i.) *P. ligularis*, ii.) *P. edulis var. edulis*, iii.)*P. edulis var. flavicarpa*, iv) *P. quadrangularis,* v.) *P. maliformis*, vi) *P. tarminiana x P. tripartita*. Peak assignments: G1 – Methyl myristate, G2 – Methyl palmitoleate, G3 – Methyl palmitate, G4 – Methyl linoleate, G5 – Methyl oleate, G6 – Methyl stearate

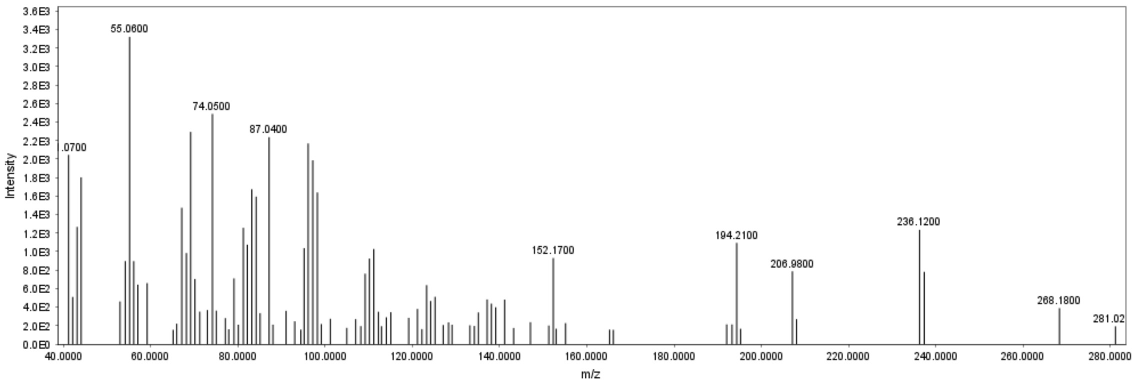

Figure S4: Peak G2 MS/MS fragmentation.

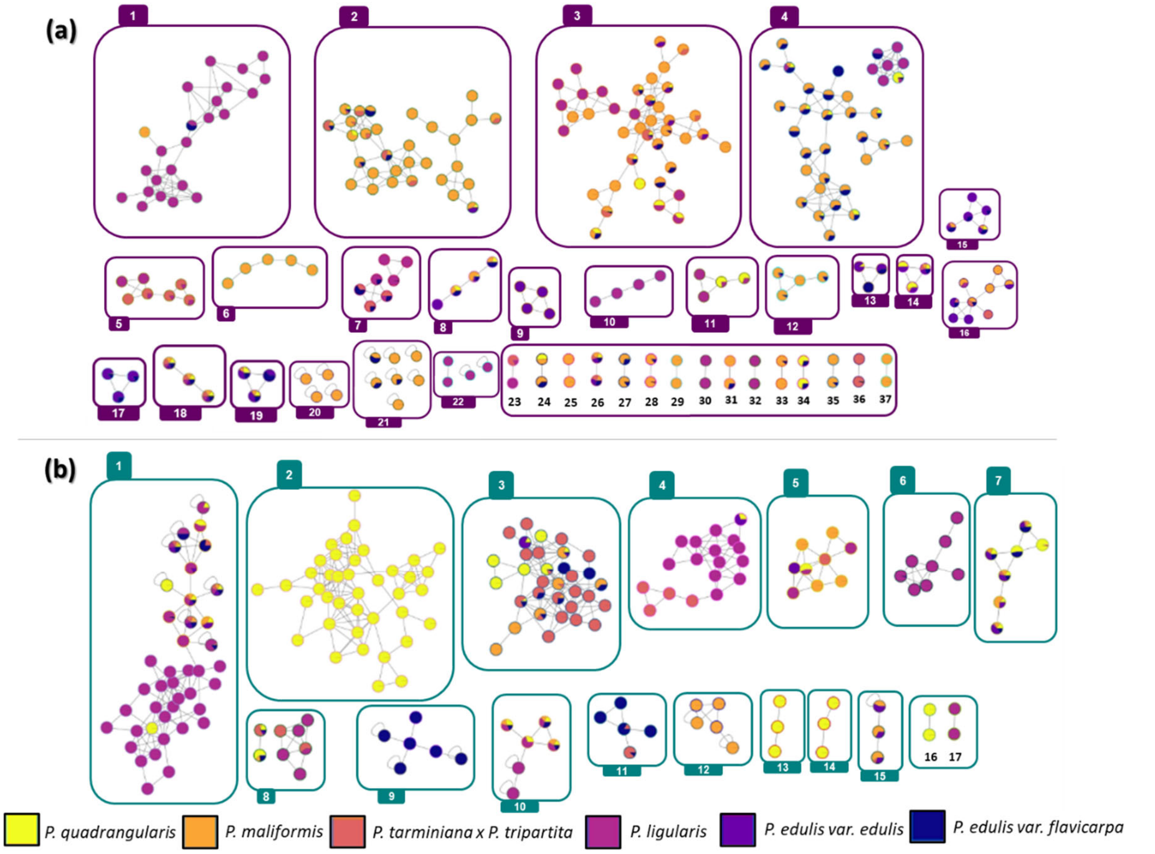

Figure S5: Most representative clusters of the molecular networks for leaves butanolic fractions in: (**a**) Negative ionization mode, according to their CF_Dparent notation: 1. Flavonoid-3-O-glycosides, 2. Flavonoid-7-O-glycosides, 3. Triterpene saponins, 4. Linoleic

acids and derivatives, 5. Biflavonoids and polyflavonoids, 6. Flavonoid 8-Cglycosides, 7. Flavones, 8. Purine nucleosides, 9. Anthraquinones, 10. Macrolactams, 11. Carbazoles, 12. Jasmonic acids, 13. Alkaloids and derivatives, 14. Polychlorinated biphenyls, 15. Phenylimidazoles, 16.Phenolic glycosides, 17. Indoles-3-acetic acid derivatives, 18. Aryl-phenylketones, 19. Phenylhydantoins, 20. Oligopeptides, 21. Cyclic depsipeptides, 22. Hybrid peptides, 23. Pyrimidinecarboxylic acids and derivatives, 24. Oxanes, 25. Saccharolipids, 26. Triterpenoids, 27. Medium chains keto acids and derivatives, 28. Pyranones and derivatives, 29. Lignan glycosides, 30. Dipeptides, 31. Terpene glycosides, 32. Eudesmanolides, 33. 1,2-diacylglicerol-3-phosohates, 34. Pyrimidones, 35. Long-chain fatty acids, 36. Gamma butyrolactones, 37. Germacranolides and derivatives; (**b**) Positive ionization mode, according to their CF_Dparent notation: 1. Macrolide lactams/ benzoic acid esters, 2. Steroidal glycosides, 3. Flavonoid O-glucuronides, 4. Proline and derivatives, 5. Benzoic acids, 6. Diterpene glycosides, 7. Bicyclic monoterpenoids, 8. Dipeptides, 9. Phenylazetidines, 10. Acyclic monoterpenoids, 11. Flavonoids- 8-C-glycosides, 12. Guanidines, 13.1,2-diacylglycerol-3-phosphates, 14.Triterpenoids, 15. Diphenylmethanes, 16. Cardiolipids, 17. Carboxylic acids esters.

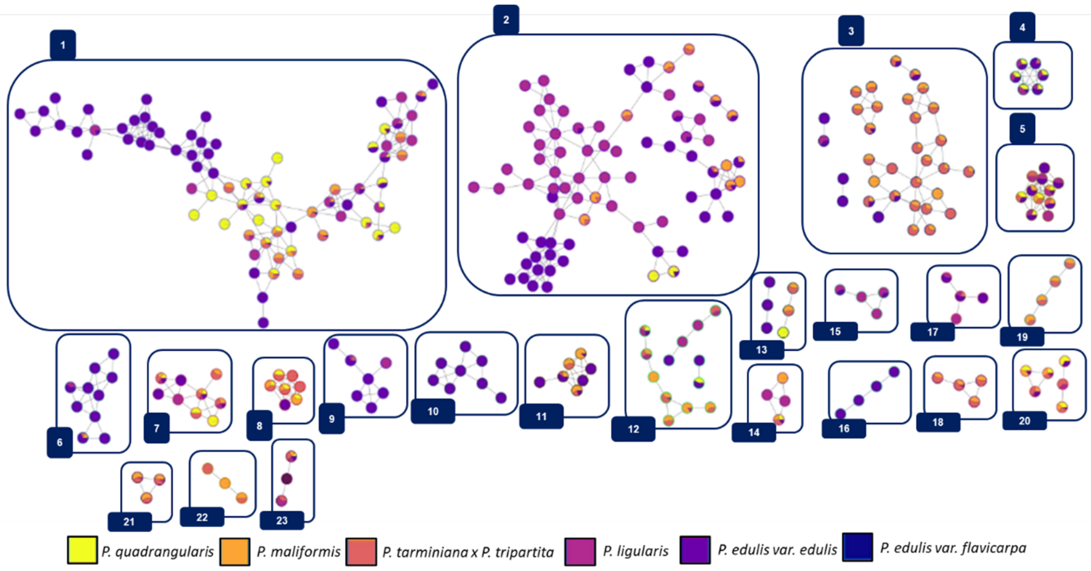

Figure S6: Most representative clusters of the molecular networks for pericarps BFs in negative ionization mode, according to their CF_Dparent notation: 1. O-glycosyl compounds, 2. Phenolic compounds, 3. Flavonoid-3-O-glycosides, 4. Long-chain fatty acids, 5. Butenolides, 6. N-acyl-alpha-hexoamines, 7. Triterpenoids, 8. Xanthones, 9. Oligosaccharides, 10. Iridoid O-glycosides, 11. Coumaric acids and derivarives, 12. Gamma butyrolactones, 13. Isoflavonoids O-glycosides, 14. Psoralens, 15. Germacranolides and derivatives, 16. Hybrid peptides, 17. 6-prenylated flavans, 18. Pyrroloindoles, 19. Tetracarboxylic acids and derivatives, 20. Xanthines, 21. Resorcinols, 22. Aryl thiophosphates, 23. Sesquiterpenoids.

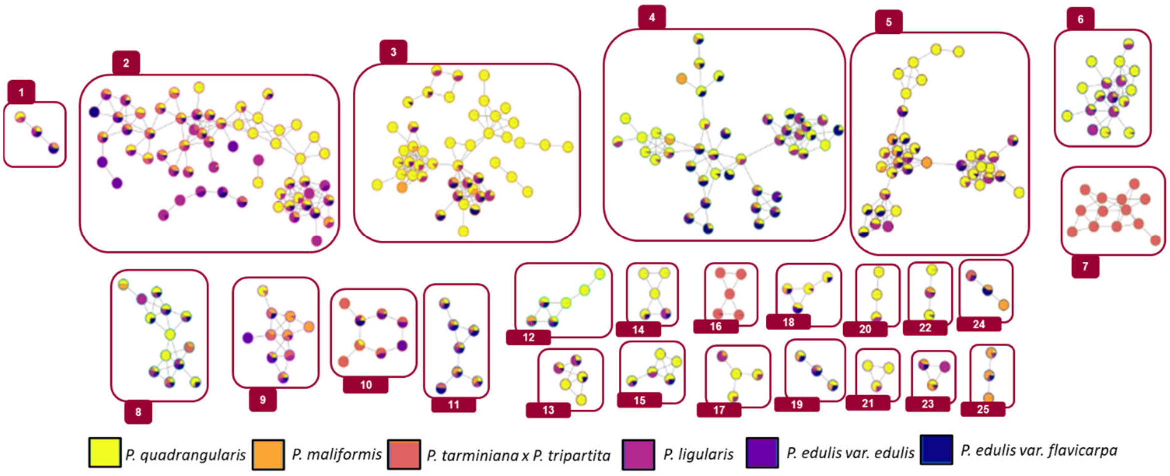

Figure S7. Most representative clusters of the molecular networks for seeds HEs in negative ionization mode, according to their CF_Dparent notation: 1. Psoralens, 2. Phenolic glycosides, 3. 2-arylbenzofuran flavonoids, 4. Triterpene saponins, 5. Triterpenoids, 6. Lignans, neolignans and related compounds, 7. Alkyl-phenylketones, 8.Germacranolides and derivatives, 9. Pentoses, 10. 2′-hydroxychalcones, 11. Linoleic acids and derivatives, 12. Flavaglines, 13. Triterpenoids, 14.N- acyl-L-alpha- aminoacids, 15. Limonoids, 16. 2′Dihydrochalcones, 17. Chlorinated dibenzo-p-dioxins, 18. Phenylpropanoic acids, 19. Long chin fatty acids, 20. Sulfanilides, 21. Oligosaccharides, 22. Benxoxepines, 23. Triterpenoids, 24. Stilbene glycosides, 25. Linoleic acids and derivatives.

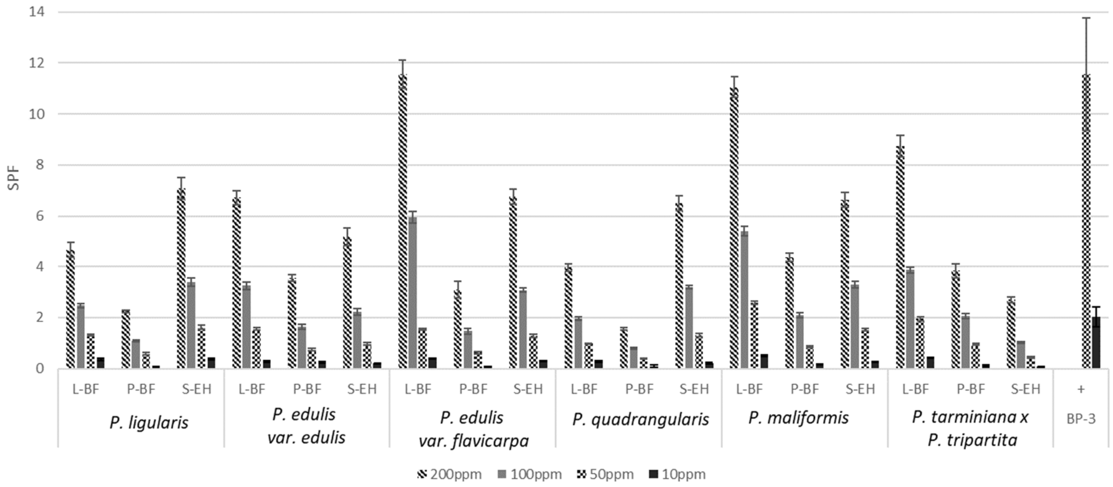

Figure S8: Sun protection factor (SPF) screening. L:Leaves; P: Pericaps; S: Seeds; BF: Butanolic fraction; HE: Hydroethanolic extract; BF-3:Benzophenone-3.

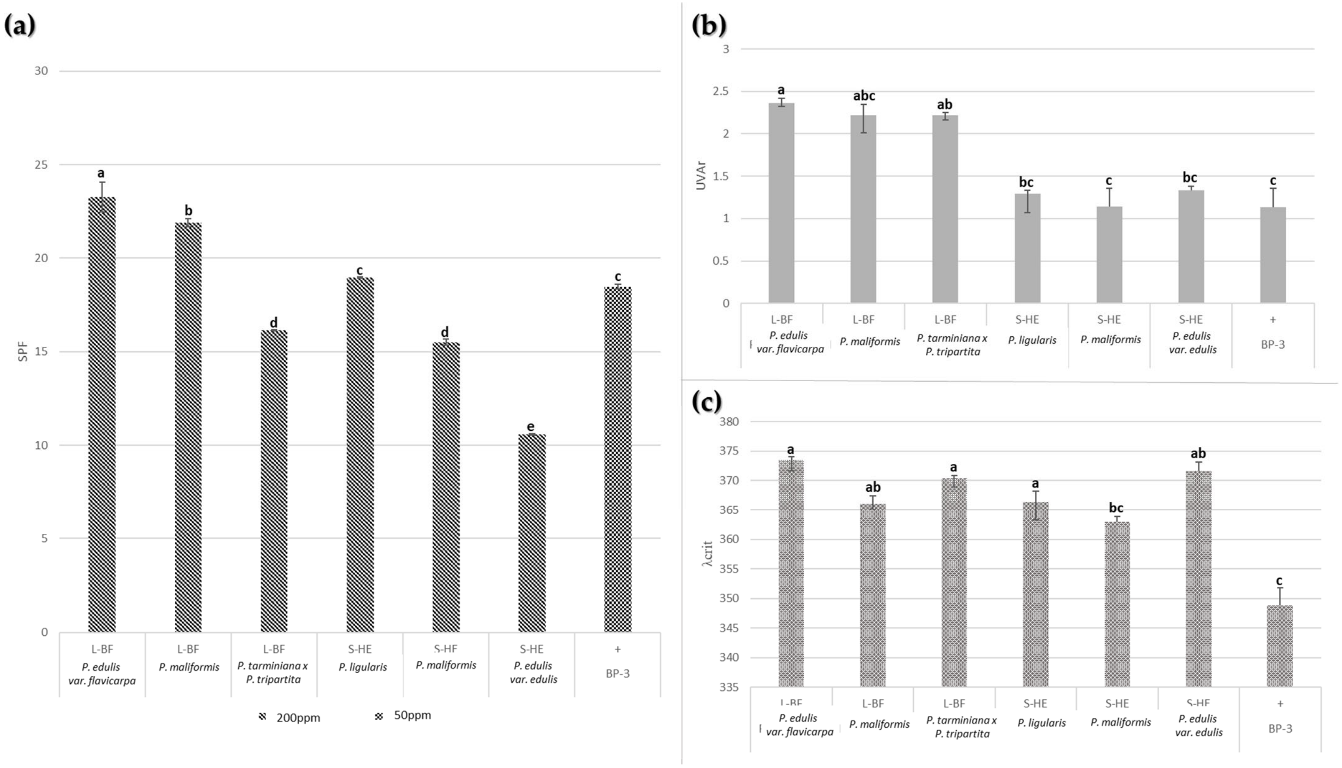

Figure S9: Photoprotection determined by spectrophotometry for the selected extracts: (a) SPF determined at different concentrations; (b) UVA ratio (UVAr); (c) critical wavelength (λ_{crit}). L:Leaves; P: Pericaps; S: Seeds; BF: Butanolic fraction; HE: Hydroethanolic extract; BF-3:Benzophenone-3. Data are expressed as mean ± SD (n = X). Different letters indicate statistically significant differences among samples according to ANOVA followed by Tukey's test ($p < 0.05$).

Table S1. Extraction yields of BF and HE extracts. BF: Butanolic Fraction; HE: Hydroethanolic Extract

Specie	Leaves BF (%)	Pericarps BF (%)	Seeds HE (%)
P. ligularis	3.3	1.4	4.5
P. edulis var. edulis	0.8	1.4	2.0
P. edulis var. flavicarpa	1.5	0.5	2.3
P. maliformis	3.0	4.5	2.5
P. quadrangularis	2.3	1.5	3.3
P. tarminiana x P. tripartita	1.2	1.2	1.6

Table S2. Fatty acids identified in the FAHOs from the evaluated *Passiflora* seeds, reported as methyl ester form (FAMEs).

Peak	RT (min)	KI[1]	FAME	Relative abundance (%)	Species[2]
G1	11.62±0.00	1727	Methyl myristate (C14:0)	1.2%	*P. quadrangularis* (1.2%)
G2	13.53±0.00	1908	Methyl palmitoleate (C16:1)[3]	1.5%	*P. quadrangularis* (1.5%)
G3	13.72±0.01	1929	Methyl palmitate (C16:0)	7.7% - 14.9%	*P. ligularis* (8.3%); *P. edulis var. edulis* (7.7%); *P. edulis var. flavicarpa* (8.8%); *P. quadrangularis* (14.9%); *P. maliformis* (10.1%); *P. tarminiana x P. tripartita* (9.8%)
G4	15.40±0.01	2099	Methyl linoleate (C18:2, ω-6)	57.1% - 79.5%	*P. ligularis* (74.6%); *P. edulis var. edulis* (79.5%); *P. edulis var. flavicarpa* (75.2%); *P. quadrangularis* (57.1%); *P. maliformis* (69.9%); *P. tarminiana x P. tripartita* (74.2%)
G5	15.44±0.01	2106	Methyl oleate (C18:1, ω-9)	10.9% - 17.6%	*P. ligularis* (15.2%); *P. edulis var. edulis* (10.9%); *P. edulis var. flavicarpa* (14.1%); *P. quadrangularis* (22.5%); *P. maliformis* (17.6%); *P. tarminiana x P. tripartita* (13.3%)
G6	15.64±0.01	2029	Methyl stearate (C18:0)	1.9% - 2.8%	*P. ligularis* (1.9%); *P. edulis var. edulis* (1.9%); *P. edulis var. flavicarpa* (1.9%); *P. quadrangularis* (2.8%); *P. maliformis* (2.4%); *P. tarminiana x P. tripartita* (2.7%)

RT: Retention time

[1] Kovats index were calculated for P. quadrangularis hexane extract using a C12–C24 n-alkane standard mixture as reference in GC-MS

[2] Species with the specific relative abundance of each fatty acid

[3] Propoused by GC-MS analysis

Table S3: Screening SPF values at 100 ppm and Tukey's multiple comparison test among samples.

Specie	Part-extract	SPF	SD	Tukey comparison[1]
P. ligularis	L-BF	2.47	0.09	f
	P-BF	1.09	0.03	i
	S-HE	3.39	0.17	d
P. edulis var. edulis	L-BF	3.25	0.15	d e
	P-BF	1.64	0.09	h
	S-HE	2.22	0.13	c
P. edulis var. flavicarpa	L-BF	5.95	0.23	a
	P-BF	1.47	0.12	e
	S-HE	3.08	0.08	d e

Specie	Part-extract	SPF	SD	Tukey comparison[1]
P. quadrangularis	L-BF	1.96	0.07	g h
	P-BF	0.80	0.04	i
	S-HE	3.20	0.06	d e
P. maliformis	L-BF	5.40	0.21	b
	P-BF	2.10	0.09	g
	S-HE	3.29	0.13	d e
P. tarminiana x P. tripartita	L-BF	3.87	0.12	c
	P-BF	2.06	0.10	g
	S-HE	1.04	0.03	i

L: Leaves; P: Pericarps; S:Seeds, BF: Butanolic fraction; HE: Hydroethanolic extract; SPF: Sun protection factor; SD: Standard Deviation

[1] Different letters indicate statistically significant differences among samples according to ANOVA followed by Tukey's test ($p < 0.05$).

Table S4: Collection data (Location and date) of the commercial *Passiflora* species studied.

Specie	Common names	Part of the plant	Cultivation site	Acquisition date	Acquisition place	Voucher
P. ligularis	Sweet granadilla /granadilla	Leaves	Anolaima (Cundinamarca)	19/08/2021	Paloquemao Marketplace (Av. Cra 30 #25-04 p.106)	COL602878
		Peels		13/09/2021	Paloquemao Marketplace (Av. Cra 30 #25-04 p.106)	
		Seeds			Paloquemao Marketplace (Av. Cra 30 #25-04 p.106)	
P. edulis var. edulis	Purple passion fruit/ Gulupa	Leaves	Cogua (Cundinamarca)	25/08/2021	OCATI®	COL530661
		Peels	Pasca (Cundinamarca)	13/09/2021	Paloquemao Marketplace (Av. Cra 30 #25-04 p.106)	
		Seeds			Paloquemao Marketplace (Av. Cra 30 #25-04 p.106)	
P. edulis var. flavicarpa	Yellow passion fruit/ Maracuyá amarillo	Leaves	Arauca	13/05/2023	Collection[a].	-
		Peels	Tolima	13/09/2021	Paloquemao Marketplace (Av. Cra 30 #25-04 p.106)	
		Seeds	Tolima		Paloquemao Marketplace (Av. Cra 30 #25-04 p.106)	
P. quadrangularis	Giant granadilla/ Badea	Leaves	Icononzo (Tolima)	28/07/2021	20 de Julio Marketplace (Cra. 6 #24ª-30 p.64)	COL589241
		Peels		13/09/2021	Paloquemao Marketplace (Av. Cra 30 #25-04 p.106)	
		Seeds		14/09/2021	Paloquemao Marketplace (Av. Cra 30 #25-04 p.106)	

Specie	Common names	Part of the plant	Cultivation site	Acquisition date	Acquisition place	Voucher
P. maliformis	Sweet calabash/ Cholupa	Leaves<br>Peels<br>Seeds	Rivera (Huila)	26/04/2023<br>5/09/2022<br>6/09/2022	Collection[a].<br>Neiva Marketplace<br>FRUCAMPS®	HPUJ 30752
P. tarminiana X P. tripartita	Banana passion fruit/ Curuba	Leaves<br>Peels<br>Seeds	Tibaná (Boyacá)	16/03/2023	Collection[a].<br>Collection[a].<br>Collection[a].	-

[a]PNM: Grupo de investigación Productos Naturales Marinos y Frutas de Colombia

Table S5: Collision energy ramp applied for ion fragmentation in MS/MS analysis.

Isolation mass value (m/z)	Charge state value (z)	Isolation width value (m/z)	Collision energy value (eV)
100	1	4	22
300	1	5	27
500	1	6	35
1000	1	8	45
2000	1	10	50
100	2	4	18
300	2	5	22
500	2	6	30
1000	2	8	35
2000	2	10	50

Table S6: Ion source parameters for LC-MS/MS analysis.

Ion source parameter	Value
Capillary voltage	4500V
Ion source temperatura	250°C
Drying gas flow	9L/min
Nebulizer gas pressure (nitrogen)	4bar

Table S7: Data processing parameters for HPLC-MS/MS analysis using MZmine and GNPS.

PARAMETER	LEAVES-FB(-)[1]	LEAVES-FB(+)[2]	PERICARPS-FB(-)[1]	SEEDS-HE(-)[1]
**Mass Detection (centroid)**				
Noise level MS1	2.00E+03	4.00E+02	1.00E+03	1.00E+03
Noise level MS2	1.00E+02	4.00E+01	5.00E+01	5.00E+01
**Chromatogram ADAP builder**				
Min group size in # of scans	4	4	4	4
Group intensity threshold	2.00E+03	4.00E+02	1.00E+03	1.00E+03
Min highest intensity	2.00E+04	4.00E+03	1.00E+04	1.00E+04
Scan to scan accuracy (m/z)	0.002 o 10ppm	0.002 o 10ppm	0.002 o 10ppm	0.002 o 10ppm
**Local minimun resolver**				
MS/MS Scan pairing<br>RT Tolerance<br>MS1 to MS2 tolerance<br>Limit by RT edges	0.2/0.1-10/1%	0.2/0.1-10/1%	0.2/0.1-10/1%	0.2/0.1-10/1%
Chromatographic threshold	90%	90%	90%	90%
Minimun search range RT	0.05	0.05	0.05	0.05
Minimum relative height	0	0	0	0
Minimum absolute height	2.00E+04	4.00E+03	1.00E+04	1.00E+04
Min ratio of peak top/edge	1.4	1.4	1.3	1.6
Peak duration range	0.01-3	0.01-3	0.01-3	0.01-3
Min # of data point	4	4	4	4
**Isotope Filter**				
m/z tolerance	0.01 o 10ppm	0.01 o 10ppm	0.01 o 10ppm	0.01 o 10ppm
Retention time tolerance (min)	0.03	0.03	0.03	0.03
Maximum charge	3	3	3	3
Monotopic shape	SI	SI	SI	SI
Never remove feature with MS2	SI	SI	SI	SI
Representative isotope	MOST INTENSE	MOST INTENSE	MOST INTENSE	MOST INTENSE
**Join aligner**				
m/z tolerance	0.004 o 10ppm	0.003 o 10ppm	0.003 o 10ppm	0.003 o 10ppm
RT tolerance	0.03/0.2	0.03/0.1	0.03/0.08	0.03/0.4
Weight m/z	3	3	3	3
Weight for RT	1	1	1	1
Compare isotope pattern<br>Isotope m/z tolerance<br>Minimum absolute intensity<br>Minimum score	SI/0.0001 o 5ppm/5E0/ 70%	SI/0.0001 o 5ppm/5E0/ 70%	SI/0.0001 o 5ppm/5E0/ 70%	SI/0.0001 o 5ppm/5E0/ 70%
**Feature list rows filters**				
Minimum features in a row	2	2	2	2
**Metacorrelate**				

RT tolerance	0.05	0.05	0.05	0.05
Min height	0	0	0	0
Intensity correlation threshold	2.00E+03	4.00E+02	1.00E+03	1.00E+03
Correlation grouping	5/2/PEARSON/85%	5/2/PEARSON/85%	5/2/PEARSON/85%	5/2/PEARSON/85%
**Ion identify networking**				
m/z tolerance	0.0015 o 5ppm	0.0015 o 5ppm	0.0015 o 5ppm	0.0015 o 5ppm
Min height	0	0	0	0
Ion identity library/Maximun charge	SI	SI	SI	SI
**GNPS**				
Precursor Ion Mass Tolerance (Da)	0.02	0.02	0.02	0.02
Fragment Ion Mass Tolerance (Da)	0.02	0.02	0.02	0.02
Min Pairs Cos	0.7	0.7	0.7	0.7
Minimum Matched Fragment Ions	6	6	6	6
Maximum shift between precursors (Da)	500	500	500	500
Network Top K	10	10	10	10
Maximum Connected Component Size (Beta)	100	100	100	100
Library Search Min Matched Peaks	6	6	6	6
Score Threshold	0.7	0.7	0.7	0.7
Search Analogs	Don't search	Don't search	Don't search	Don't search
Maximum Analog Search Mass Difference (Da)	100	100	100	100
Top results to report per query	1	1	1	1

[1]Negative ionization mode
[2]Positive ionization mode

Table S8: Access links to workflows performed in GNPS for each analyzed group.

Organ/ part	Ionization mode	Workflow	Access link
Leaves	Negative	FBMN	https://gnps.ucsd.edu/ProteoSAFe/status.jsp?task=9eb196b8ac024e16902f567f421e430b
Leaves	Positive	FBMN	https://gnps.ucsd.edu/ProteoSAFe/status.jsp?task=9f7eaf9b88364c199541ff3cf859e856
Pericarps	Negative	FBMN	https://gnps.ucsd.edu/ProteoSAFe/status.jsp?task=5bf5f8baa5aa4e8eb5fa1636f8dafbd1
Seeds	Negative	FBMN	https://gnps.ucsd.edu/ProteoSAFe/status.jsp?task=ad5bffc4cf4946e0a6c5252a00a661a6
Leaves	Negative	MolNetEnhacer	https://gnps.ucsd.edu/ProteoSAFe/status.jsp?task=36947fbcedef4b83b3d7280d30e99501

Organ/ part	Ionization mode	Workflow	Access link
Leaves	Positive	MolNetEnhacer	https://gnps.ucsd.edu/ProteoSAFe/status.jsp?task=28db3c2fc2094264a4f80608b657c96b
Pericarps	Negative	MolNetEnhacer	https://gnps.ucsd.edu/ProteoSAFe/status.jsp?task=def50e7a5fad449592558b7faac239e1
Seeds	Negative	MolNetEnhacer	https://gnps.ucsd.edu/ProteoSAFe/status.jsp?task=fba9ee38c8914b6587989d7f19a69f93

FBMN: Feature based molecular networking

Table S9: EE(λ) × I(λ) value established for each evaluated wavelength. EE(λ): the erythemal effect spectrum; I(λ): the solar intensity spectrum

Wavelength (λ) (nm)	EE(λ) X I(λ)
**290**	0.0150
**295**	0.0817
**300**	0.2874
**305**	0.3278
**310**	0.1864
**315**	0.0839
**320**	0.0180
